# TDC-2: Multimodal Foundation for Therapeutic Science

**DOI:** 10.1101/2024.06.12.598655

**Published:** 2024-06-21

**Authors:** Alejandro Velez-Arce, Kexin Huang, Michelle M. Li, Xiang Lin, Wenhao Gao, Tianfan Fu, Manolis Kellis, Bradley L. Pentelute, Marinka Zitnik

**Affiliations:** 1 Harvard; 2 Stanford; 3 MIT; 4 Rensselaer Polytechnic Institute

## Abstract

Therapeutics Data Commons (tdcommons.ai) is an open science initiative with unified datasets, AI models, and benchmarks to support research across therapeutic modalities and drug discovery and development stages. The Commons 2.0 (TDC-2) is a comprehensive overhaul of Therapeutic Data Commons to catalyze research in multimodal models for drug discovery by unifying single-cell biology of diseases, biochemistry of molecules, and effects of drugs through multimodal datasets, AI-powered API endpoints, new multimodal tasks and model frameworks, and comprehensive benchmarks. TDC-2 introduces over 1,000 multimodal datasets spanning approximately 85 million cells, pre-calculated embeddings from 5 state-of-the-art single-cell models, and a biomedical knowledge graph. TDC-2 drastically expands the coverage of ML tasks across therapeutic pipelines and 10+ new modalities, spanning but not limited to single-cell gene expression data, clinical trial data, peptide sequence data, peptidomimetics protein-peptide interaction data regarding newly discovered ligands derived from AS-MS spectroscopy, novel 3D structural data for proteins, and cell-type-specific protein-protein interaction networks at single-cell resolution. TDC-2 introduces multimodal data access under an API-first design using the model-view-controller paradigm. TDC-2 introduces 7 novel ML tasks with fine-grained biological contexts: contextualized drug-target identification, single-cell chemical/genetic perturbation response prediction, protein-peptide binding affinity prediction task, and clinical trial outcome prediction task, which introduce antigen-processing-pathway-specific, cell-type-specific, peptide-specific, and patient-specific biological contexts. TDC-2 also releases benchmarks evaluating 15+ state-of-the-art models across 5+ new learning tasks evaluating models on diverse biological contexts and sampling approaches. Among these, TDC-2 provides the first benchmark for context-specific learning. TDC-2, to our knowledge, is also the first to introduce a protein-peptide binding interaction benchmark.

## 1 Introduction

Biomedical machine learning (ML) faces challenges in developing versatile models that support a broad range of tasks in the realm of out-of-distribution (OOD) generalization [23, 24], and multimodal models that can incorporate effects of drugs, often organic molecules (chemistry), their interactions with proteins (targets) that trigger perturbations of biological pathways (networks) and produce phenotypic effects that can be measured in, for example, cell-based assays (single cells) before delivery to clinics (patients) [25]. These challenges are compounded by the lack of unified datasets organized across these five levels of increasing complexity based on the steps of drug discovery. Therapeutics Data Commons (TDC-1) [1, 26] addresses these challenges by providing a unified platform that consolidates therapeutic datasets, AI models, and benchmarks and facilitates a holistic approach to multimodal model development and evaluation, to facilitate algorithmic and scientific advances in therapeutics. TDC-1 had over 145,000 PyPI package installations and datasets, which achieved over 350,000 downloads, demonstrating TDC’s impact on research and development for machine learning models in therapeutics.

Models capable of accurate out-of-distribution predictions promise to expand to the vast molecular space, *whose size is estimated at* 10^6^ *potential drug-like molecules* [27], *yet less than* 10^5^ *of those are FDA-approved drugs* [28], suggesting the potential for advanced computational methods to navigate the molecular space and help find, generate and optimize candidate drugs. Further, handling multimodal data is essential for building foundation models that accurately capture the complex interactions within biological systems [29], which is vital for understanding disease mechanisms and discovering effective treatments. Further, the need for API-first data access and toolified ML models stems from the demand for flexible, scalable, and accessible models that can integrate into emerging tool-based LLMs [30, 16] and agentic workflows [31]. TDC-2 takes on these challenges by introducing a multimodal retrieval API with an API-first-dataset model. This new feature allows TDC-2 to enhance LLM workflows with capabilities such as: continual learning [30, 17, 31], dynamic contextual learning [16, 17], and integration with agents [17, 30].

Due to the inherent complexity and diversity of biomedical data, existing datasets and benchmarking efforts in drug discovery often fail to address these challenges. Benchmarks tailored to measuring the effectiveness of models at out-of-distribution predictions are rare for several key biological tasks [32]. Most dataset and benchmark providers also struggle to evaluate models using longitudinal data [8] and real-world evidence [9] due to challenges in continual data collection [33]. API integration for research workflows presents challenges in data standardization and harmonization [34], reproducibility and reliability [35], and scalability and performance [36]. Many platforms focus on specific types of data or stages of the drug development process, lacking the comprehensive framework to develop benchmarks [37] and robust evaluation metrics [38].

### Present work.

**Figure 1:**
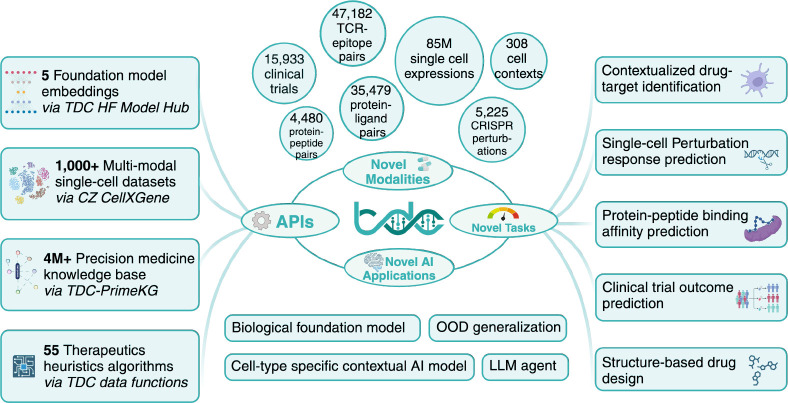
Overview of TDC-2. TDC-2 introduces an API-first Multimodal Retrieval API powering ML-task-driven [1] datasets [2, 3, 4, 5, 6, 7, 8, 9, 10, 11]and benchmarks spanning 10+ new modalities and 5 broad state-of-the-art machine learning tasks (7 total detailed in Section 4.4). The API-first design of TDC-2, built on the Model-View-Controller (MVC) [12, 13, 14] (Section 3.2) paradigm and a Domain-Specific Language (DSL) [15] (Section 3.2), unifies diverse data sources and modalities and is essential to enable integration of TDC-2 with LLMs and foundation models [16, 17, 18]. Model benchmarks highlighting biomedical AI challenges in OOD Generalization [19, 20, 21, 8] and evaluation [3, 22] of cell-type-specific contextual AI models are introduced.

The Commons 2.0 (TDC-2) aims to catalyze research in multimodal models and foundation models by integrating data, ML tasks, and benchmarks across five levels of chemistry, targets, networks, single cells, and patients. This is achieved under an API-first approach with a fine-tuning paradigm. TDC-2 (Figure 1) provides multimodal datasets, state-of-the-art pre-calculated embeddings, a comprehensive biomedical knowledge graph, and API endpoints. TDC-2 distinguishes itself by introducing an API-first [39, 40] framework that unifies data sources through a Model-View-Controller (MVC) [12] paradigm and a Domain-Specific Language (DSL) [15]. TDC-2 presents 7 novel ML tasks with fine-grained biological contexts. Three tasks introduce cell-type-specific biological context: drug-target identification [3] and chemical/genetic perturbation response prediction [19, 20]. TDC-2 introduces a protein-peptide binding affinity prediction task [9] and clinical trial outcome prediction task [8], providing tasks across antigen-processing-pathway contexts, cell type contexts, and patient contexts.

TDC-2 is designed to support ML research in some of the most pressing challenges, including but not limited to cell-type-specific ML modeling [3], inferential gap in precision medicine [41], negative-sampling challenges in peptidomimetics [22], and OOD generalization in perturbations [42, 43]. TDC-2 is focused on providing functionality to support therapeutic foundation model research. Last, exposing TDC-2 services through RESTful and RPC APIs implemented on web serves and packaged containers can help tool-based LLM systems leverage TDC-2 more effectively.

## 2 Related Work

### TDC-1, related benchmarks, and therapeutic initiatives.

Therapeutics Data Commons (TDC-1) was the first unifying platform providing systematic access and evaluation for machine learning across the entire range of therapeutics [1]. TDC-1 included 66 AI-ready datasets in the Harvard Dataverse [44]. These datasets were spread across 22 learning tasks, spanning the discovery and development of safe and effective medicines. TDC-1 also provided an ecosystem of tools and community resources, including 33 data functions and types of meaningful data splits, 23 strategies for systematic model evaluation, 17 molecule generation oracles, and 29 public leaderboards. TDC-2 augmented the biomedical modalities covered by TDC-1 data, tasks, and benchmarks to lay the foundations for building and evaluating foundation models. The Commons (TDC-2) distinguishes itself from related datasets [45, 46], benchmarks [47, 48, 49, 50], model development frameworks [51, 52], and therapeutic initiatives [53] in its more extensive coverage of relevant and robust therapeutic datasets, benchmarks, pipelines, and modalities. It also distinguishes itself via an API-first, unified platform approach to data and model retrieval, harmonization, and development.

### Emerging area of foundation models.

TDC-2 supports various prediction and generative tasks by providing curated datasets, benchmarks, and leaderboards. Additionally, recent advancements in LLM agents, such as Toolformer [30] ChatNT [54], GeneGPT [55], Gorilla [16], ToolLLM [56], CRAFT [57], and RestGPT [58] showcase the potential of integrating API tools to allow these systems to call external functions and APIs. Models like AlphaFold [29], Evo [59], and ESM [60] highlight the complementary nature of sequence- and structure-based approaches. Integrating multimodal learning approaches may be essential in capturing the full complexity of gene function [61]. The API-first [40, 39, 62] approach adopted by TDC-2’s multimodal retrieval API enables seamless integration of extensive resources with advanced models, accelerating the development of therapeutic foundation models.

## 3 TDC-2’s Multimodal Datasets and Model Retrieval API

### 3.1 Overview of TDC-2

The Commons 2.0 (TDC-2) integrates single-cell biology of diseases, biochemistry of molecules, and drug effects through an extensive array of multimodal datasets, AI-powered API endpoints, innovative multimodal tasks and model frameworks, and comprehensive benchmarks.

#### New modalities.

TDC-2 introduces over 1,000 multimodal datasets covering approximately 85 million cells [53]. These datasets include pre-calculated embeddings from five state-of-the-art machine learning models, large-scale single-cell atlases and datasets, and a biomedical knowledge graph detailing 17,080 diseases and 4,050,249 relationships [63]. TDC-2 broadens the scope of machine learning tasks across therapeutic pipelines and more than 10 new modalities. These include single-cell gene expression data, clinical trial data, peptide sequence data, peptidomimetics protein-peptide interaction data from AS-MS spectroscopy, novel 3D structural protein data, and cell-type-specific protein-protein interaction networks at single-cell resolution. These tasks encompass datasets with 32 CRISPR perturbations, nine drug-based perturbations, and drug-target interaction data for two diseases across 156 cell-type-specific contexts.

#### Innovative API-first-dataset design.

The API-first design of TDC-2, built on the Model-View-Controller (MVC) [12] paradigm and a Domain-Specific Language (DSL) [15], unifies diverse data sources and modalities. The API-first-dataset design is essential to enable integration of TDC-2 with LLMs for in-context learning [16, 17, 18], facilitating dynamic data access, ensuring real-time updates, and enhancing the accuracy and relevance of responses.

#### Novel ML tasks and therapeutic pipelines.

TDC-2 introduces three new learning tasks focusing on cell-type-specific biological contexts, drug-target identification [3], and prediction of responses to chemical and genetic perturbations [20, 19, 42]. TDC-2 is the first renowned multimodal open-source dataset and benchmark provider to introduce a protein-peptide binding affinity prediction task [9] and a precision-medicine-oriented clinical trial outcome prediction task [8].

#### Benchmarking and model evaluation.

TDC-2 provides benchmarks for over 15 state-of-the-art models across more than five new learning tasks. These are tailored to take on some of the most pressing machine learning challenges in biomedicine, including but not limited to cell-type-specific machine learning modeling and evaluation [3], the inferential gap in precision medicine [41], negative-sampling challenges in peptidomimetics [22], and out-of-distribution model generalizability across unseen cell lines and perturbations [42, 43].

#### AI-powered endpoints.

Through The Commons’ Model Hub and CZ CellXGene [53], TDC-2 offers API endpoints with multimodal retrieval capabilities. These endpoints provide access to protein embeddings under specific biological contexts and model predictions.

### 3.2 TDC-2 Model-View-Controller Design

TDC-2 drastically expands dataset retrieval capabilities available in TDC-1 beyond those of other leading benchmarks. Leading benchmarks, like MoleculeNet [46] and TorchDrug [47] have traditionally provided dataloaders to access file dumps. TDC-2 introduces API-integrated multimodal data-views [12, 64, 14]. The software architecture of TDC-2 was redesigned using the Model-View-Controller (MVC) design pattern [13, 65] (Section 3.2). The MVC architecture separates the model (data logic), view (UI logic), and controller (input logic), which allows for the integration of heterogeneous data sources and ensures consistency in data views [12]. The MVC pattern supports the integration of multiple data modalities by using data mappings and views [14]. The MVC-enabled-multimodal retrieval API is powered by TDC-2’s Resource Model (Section 3.3).

#### TDC DataLoader (*Model*).

Per the TDC-1 specification, this component queries the underlying data source to provide raw or processed data to upstream function calls. We augmented this component beyond TDC-1 functionality to allow for querying datasets introduced in TDC-2, such as the CZ CellXGene.

#### TDC meaningful data splits and multimodal data processing (*View*).

Per the TDC-1 specification, this component implements data splits to evaluate model generalizability to out-of-distribution samples and data processing functions for multiple modalities. We augmented this component to act on data views [12] specified by TDC-2’s controller.

#### TDC-2 Domain-Specific Language (*Controller*).

TDC-2 develops an Application-Embedded Domain-Specific Data Definition Programming Language facilitating the integration of multiple modalities by generating data views from a mapping of various datasets and functions for transformations, integration, and multimodal enhancements while maintaining a high level of abstraction [15] for the Resource framework. We include examples of developing multimodal datasets leveraging this MVC DSL in Appendix A.2.1.

### 3.3 TDC-2 Resource Model

The Commons introduces a redesign of TDC-1’s dataset layer into a new data model dubbed the TDC-2 resource, developed under the MVC paradigm to integrate multiple modalities into the API-first model of TDC-2.

#### CZ CellXGene with single-cell biology datasets.

CZ CellXGene [53] is an open-source platform for single-cell RNA sequencing data analysis. We leverage the CZ CellXGene to develop a TDC-2 Resource Model for constructing large-scale single-cell datasets that maps gene expression profiles of individual cells across tissues, healthy and disease states. TDC-2 leverages the SOMA (Stack of Matrices, Annotated) API, adopts TileDB-SOMA [66] for modeling sets of 2D annotated matrices with measurements of features across observations and enables memory-efficient querying of single-cell modalities (i.e., scRNA-seq, snRNA-seq), across healthy and diseased samples, with tabular annotations of cells, samples, and patients the samples come from.

We develop a remote procedure call (RPC) API taking the string name (e.g., Appendix A.2.2) of the desired reference dataset as specified in the CellXGene [53]. The remote procedure call for fetching data is defined as a Python generator expression, allowing the user to iterate over the constructed single-cell atlas without loading it into memory [67]. Specifying the RPC as a Python generator expression allows us to use memory-efficient querying as provided by TileDB [66]. The single-cell datasets can be integrated with therapeutics ML workflows in TDC-2 using tools such as PyTorch’s IterableDataset module [68].

#### Knowledge graph, external APIs, and model hub.

We have developed a framework for biomedical knowledge graphs to enhance the multimodality of dataset retrieval via TDC-2’s Resource Model. Our system leverages PrimeKG to integrate 20 high-quality resources to describe 17,080 diseases with 4,050,249 relationships [63]. Our framework also extends to external APIs, with data views currently leveraging BioPython [69], for obtaining nucleotide sequence information for a given non-coding RNA ID from NCBI [69], and The Uniprot Consortium’s RESTful GET API [70] for obtaining amino acid sequences. In addition, we’ve developed a framework that allows access to embedding models under diverse biological contexts via the TDC-2 Model Hub. Examples using these components are in Appendix A.2.4 A.2.3.

## 4 TDC-2 Tasks, Datasets, and Benchmarks with Results

TDC-2 drastically expands TDC-1’s ML tasks and benchmarks across pipelines and modalities. It presents novel contextualized learning tasks at single-cell resolution, including drug-target identification and counterfactual predictions for drug and CRISPR-based interventions. It also introduces peptide-based tasks, including protein-peptide and TCR-epitope binding affinity prediction tasks. The complete formulation of tasks, including datasets and benchmark results, is included in the Appendix. We introduce clinical trial outcome prediction and structure-based drug design, both formulated in Appendix A.3.4 A.4.

### 4.1 TDC.scDTI: Contextualized Drug-Target Identification

#### Motivation.

Single-cell data have enabled the study of gene expression and function at the level of individual cells across healthy and disease states [71, 53, 61]. To facilitate biological discoveries using single-cell data, machine-learning models have been developed to capture the complex, cell-type-specific behavior of genes [72, 73, 74, 3]. In addition to providing the single-cell measurements and foundation models, TDC-2 supports the development of contextual AI models to nominate therapeutic targets in a cell type-specific manner [3]. We introduce a benchmark dataset, model, and leaderboard for context-specific therapeutic target prioritization, encouraging the innovation of model architectures (e.g., to incorporate new modalities, such as protein structure and sequences [75, 76, 77, 78, 79], genetic perturbation data [80, 81, 82, 83], disease-specific single-cell atlases [84, 85, 86], and protein networks [87, 88, 89]). TDC-2’s release of TDC.scDTI is a significant step in standardizing benchmarks for more comprehensive assessments of context-specific model performance.

#### Task definition: Contextualized drug-target identification.

*The goal is to train a model* $f_{\theta }$ *for predicting the probability* $\overset{ˆ}{y}\in [0,\;1]$ that a protein is a candidate therapeutic target in a specific cell type. The model learns an estimator for a function of a protein target t∈T and a cell-type-specific biological context c∈C *as input, and the model is tasked to predict:* $\overset{ˆ}{y}=f_{\theta }(t\in T,\;c\in C)$.

#### Dataset and benchmark.

We use curated therapeutic target labels from the Open Targets Platform [4] for rheumatoid arthritis (RA) and inflammatory bowel disease (IBD) [3]. Further details on the composition of this dataset are in Appendix A.3.1. We benchmark PINNACLE [3]—trained on cell type specific protein-protein interaction networks—and a graph attention neural network (GAT) [90]—trained on a context-free reference protein-protein interaction network—on the curated therapeutic targets dataset. As expected, PINNACLE underperforms when evaluated on context-agnostic metrics (Table 1) and drastically outperforms GAT when evaluated on context-specific metrics (Appendix Table 1). Appendix A.3.1 shares further evidence the most predictive cell type contexts identified by PINNACLE are most relevant to each disease [3] (Appendix Figure 2).

To our knowledge, TDC-2 provides the first benchmark for context-specific learning [91]. TDC-2’s contribution helps standardize the evaluation of single-cell ML models for drug target identification and other single-cell tasks [74, 72, 3, 73].

### 4.2 TDC.PerturbOutcome: Perturbation-Response Prediction

#### Motivation.

Predicting gene expression responses to genetic or chemical perturbations is crucial in systems biology and precision medicine. This task involves forecasting the changes in gene expression profiles in response to specific perturbations applied to a biological system, such as gene knockouts, knockdowns, overexpression, or chemical treatments. Despite advancements in model development for perturbation outcome prediction, generalizing to unseen perturbations and cell lines remains a challenge in predicting gene expression responses. While several innovative approaches, such as deep generative models [92] [43], compositional autoencoders [21], and active learning and sequential design [93], have been proposed, they each have limitations. Most models cannot generalize to perturbations that were not seen during model training.

While models like GEARS [19] and chemCPA [20] showed great promise in generalizing to unseen perturbations, they do not generalize to unseen cell lines. Furthermore, both GEARS and chemCPA are limited to genetic and chemical perturbations, respectively. While approaches like PerturbNet [43] and Biolord [42] can generalize across chemical and genetic perturbations, they still struggle to generalize across cell lines and biological contexts. Without modifications, Biolord is unable to generalize to unseen perturbations. TDC-2 takes on this challenge by introducing a model framework, task definition, and a benchmark for the Perturbation-Response Prediction task to enable ML research in foundation models for comprehensive *in silico* perturbation modeling across biological contexts, chemical and genetic perturbations, and seen and unseen perturbations.

#### Task definition: Perturbation-response prediction.

*The Perturbation-Response Prediction learning task is to learn a regression model* $f_{\theta }$ *estimating the perturbation-response gene expression vector* $\overset{ˆ}{\vec{e_{1}}}$ *for a perturbation applied in a cell-type-specific biological context to a control. The model learns an estimator for a function taking control cell gene expression* $\vec{e_{0}}\in E_{⊬}$, a perturbation p∈P. Their cell-type-specific biological context c∈C, and the model is tasked to generate: $\overset{ˆ}{\vec{e_{1}}}=f_{\theta }(p\in P,\;\vec{e_{0}}\in E_{⊬},\;c\in C)$.

#### Dataset and benchmark.

In TDC-2, we’ve used the scPerturb [2] datasets to benchmark the Perturbation-response prediction model generalizability across seen/unseen perturbations and cell lines. We benchmark models in genetic and chemical perturbations using metrics measuring intra/inter-cell line and seen/unseen perturbation generalizability. We provide results measuring unseen perturbation generalizability for Gene Perturbation Response Prediction using the scPerturb gene datasets (Norman K562, Replogle K562, Replogle RPE1). For Chemical Perturbation Prediction, we’ve evaluated chemCPA utilizing cold splits on perturbation type and show a significant decrease in performance for 3 of 4 perturbations evaluated. We’ve also included Biolord [42] and scGen [92] for comparison. These tests were run on sciPlex2 [2].

#### Genetic perturbation response prediction.

Results for different scenarios are in Appendix A.3.2.

#### Chemical perturbation response prediction.

**Figure 3:**
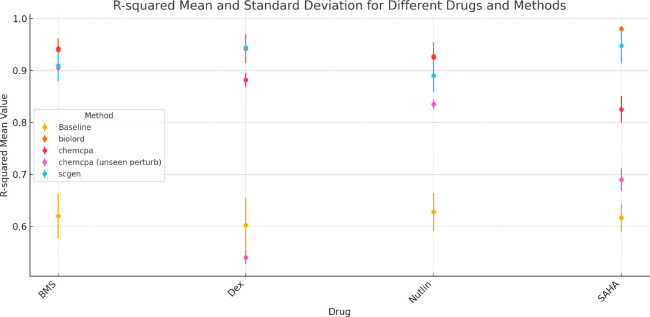
R-squared of chemical perturbation predictions across models and drug types. We include results for chemCPA when tested for unseen perturbation. The performance of chemCPA dropped significantly for Dex, Nutlin, and SAHA.

The dataset used was 4 drug-based perturbations from sciPlex2 [2] (BMS, Dex, Nutlin, SAHA). Results are shown in Table 2 and Figure 3. chemCPA’s performance dropped by an average of 15% across the 4 perturbations. The maximum drop was 34%. Code for intra/inter cell-line benchmarks for chemical (drug) and genetic (CRISPR) perturbations is in Appendix B.1 and Appendix B.1, respectively. Using this code, users can evaluate models of their choice on the benchmark and submit them to the TDC-2 leaderboards for this task (Appendix B.2).

### 4.3 TDC.ProteinPeptide: Protein-Peptide Interaction Prediction

#### Motivation.

Protein-peptide interactions differ significantly from protein-protein interactions. Predicting binding affinity for peptides is more complex than for proteins due to their flexibility and ability to adopt multiple conformations [94, 95]. High-quality binding affinity data for protein-protein interactions are more readily available than for protein-peptide interactions [96]. The heterogeneity of peptides also leads to a diverse range of binding modes and affinities [32]. Predictive models for protein-peptide interactions must consider peptide flexibility and sequence variability, leading to more complex and computationally intensive approaches [97]. Evaluating protein-peptide binding prediction models requires standardized benchmarks, presenting challenges in assessing and validating model performance across different studies [32].

Despite the availability of several benchmarks for protein-protein interactions, this is not the case for protein-peptide binding affinity prediction. The renowned multi-task benchmark for Protein sEquence undERstanding (PEER) [49] and MoleculeNet [46] both lack support for a protein-peptide binding affinity prediction task. MoleculeNet defines a single general Protein-Ligand binding affinity task, which TDC-2 also includes, and is limited in its supported data modalities [5]. Approaches relying solely on the sequence and structural data tend not to be as accurate in applications (i.e.,., predicting the affinity of peptides to MHC class II [98]) those integrating additional modalities, such as information about prior steps in the biological antigen presentation pathway [99]. Furthermore, protein-peptide binding mechanisms vary wildly by cellular and biological context [100, 101, 102, 103]. SoTA models, as such, tend to be restricted to one task instance (i.e., T Cell Receptor (TCR) and Peptide-MHC Complex or B Cell Receptor (BCR) and Antigen Peptides binding) and don’t span protein-peptide interactions [104, 105, 106, 107, 108, 109, 110].

TDC-2 introduces a model framework, task definition, datasets, and benchmarks for the Protein-Peptide Interaction Prediction task. It evaluates the model’s generalizability to newly discovered peptides and highlights negative sampling challenges.

#### Task definition: Protein-peptide interaction prediction.

*The Protein-Peptide Interaction Prediction learning task is to learn a binary classification model* $f_{\theta }$ *estimating the probability*, $\overset{ˆ}{y}$, of a protein-peptide interaction meeting specific biomarkers. The model learns an estimator for a function taking a target protein p∈P, a peptide candidate s∈S, an antigen processing pathway profile a∈A, an interaction set i∈I, and a cell-type-specific biological context c∈C *as inputs, and the model is tasked to predict:* $\overset{ˆ}{y}=f_{\theta }(p\in P,\;s\in S,a\in A,i\in I,c\in C)$.

#### 4.3.1 Datasets and Benchmarks

##### TCR-Epitope (Peptide-MHC Complex) interaction prediction.

The critical challenge in TCR-Epitope (Peptide-MHC Complex) Interaction Prediction lies in creating a model that can effectively generalize to unseen TCRs and epitopes [111]. While TCR-H [112] and TEINet [113] have shown improved performance on prediction for known epitopes, by incorporating advanced features like attention mechanisms and transfer learning, the performance significantly drops for unseen epitopes [114, 115]. Another challenge in TCR-Epitope Interaction Prediction lies in the choice of method for negative sampling, with non-binders often underrepresented or biased in curated datasets, leading to inaccurate predictions when generalized [22].

TDC-2 establishes a curated dataset and benchmark within its Protein-Peptide Binding Affinity prediction task to take on both model generalizability to unseen TCRs and epitopes and model sensitivity to negative sampling methodology. Benchmarking datasets use three types of negative sampling methods: random shuffling of epitope and TCR sequences (RN), experimental negatives (NA), and pairing external TCR sequences with epitope sequences (ET). We harness data from the TC-hard dataset [7] for the first two types and PanPep [6] for the third type. Both datasets use hard [7] splits, ensuring that epitopes in the testing set are not present in the training set. Our results (Table 3) show the lack of a reasonable negative sampling method, with model performance evaluation shown to be unsatisfactory. For two sampling methods, all models perform poorly. The best-performing model in ET is MIX-TPI, with roughly 0.70 AUROC. The best-performing model in RN is AVIB-TCR, with approximately 0.576 AUROC. For NA, 4 of 6 models perform near-perfectly as measured on AUROC.

Models benchmarked include AVIB-TCR [116], MIX-TPI [117], Net-TCR2 [118], PanPep [111], TEINet [113], and TITAN [115]. Results are available in Table 3.

#### AS-MS data for newly discovered ligands - Protein-peptide binding affinity prediction.

To benchmark future generalized protein-peptide models for this task, we use affinity selection-mass spectrometry data which identified ligands binding to single biomolecular targets (MDM2, ACE2, Anti-HA 12CA5) [119, 9]. Further details on this dataset are included in the Appendix A.3.3.

### 4.4 Other New ML Tasks Introduced in TDC-2: TDC.TrialOutcome and TDC.SBDD

#### Clinical trial outcome prediction.

TDC-2 introduces a model framework, task definition, dataset, and benchmark for the Clinical Outcome Prediction task tailored to precision medicine. The framework and definition aim to assess clinical trials systematically and comprehensively by predicting various endpoints for patient sub-populations. Our benchmark uses the Trial Outcome Prediction (TOP) dataset [8]. TOP consists of 17,538 clinical trials with 13,880 small-molecule drugs and 5,335 diseases. We include the task formulation and dataset details in the Appendix. Benchmark details are in Appendix A.3.4. Code for reproducing experiments can be found in Appendix A.3.4.

#### Structure-based drug design tasks.

Structure-based drug design aims to create diverse new molecules that bind to protein pockets (3D structures) and have favorable chemical properties. These attributes are evaluated using pharmaceutically-relevant oracle functions. In this task, an ML model learns molecular traits of protein pockets from a comprehensive dataset of protein-ligand pairs. Subsequently, potential new molecules can be generated using the acquired conditional distribution. The generated molecules must exhibit outstanding properties, including high binding effectiveness and structural variety. They must meet other user-specified criteria, such as the feasibility of synthesis (synthesizability/designability) and similarity to known drugs. Our task consists of multiple components, which we formulate in the Appendix. We detail datasets [5, 10, 11] in Appendix A.4.

## 5 Conclusion

TDC-2 introduces an API-first architecture for maximal compatibility with tool-based LLMs and agents, such as [30, 16, 120] and many other emerging systems. It does so via the development of a multimodal data and model retrieval API leveraging the Model-View-Controller [12, 65, 13] paradigm to introduce data views [14] and a domain-specific-language [15].

TDC-2 drastically expands the modalities and therapeutic pipelines previously available on TDC-1 [1, 26]. TDC-2 supports a far larger set of data modalities and ML tasks than other datasets [53] and benchmarks [47, 48, 49, 46]. Modalities in TDC-2 include but are not limited to: single-cell gene expression atlases [53, 71], chemical and genetic perturbations [2], clinical trial data [8], peptide sequence data [7, 6], peptidomimetics protein-peptide interaction data from AS-MS spectroscopy [9, 119], novel 3D structural protein data [5, 11, 10], and cell-type-specific protein-protein interaction networks at single-cell resolution [3]. TDC-2 introduces ML tasks taking on open challenges, including the inferential gap in precision medicine [121, 41], model generalizability across cell lines [19, 42] and single-cell perturbations [20] that were not encountered during model training, and evaluation of models across a broad range of diverse biological contexts [3, 22].

## Figures and Tables

**Table 1:** Cell-type specific target nomination for 2 therapeutic areas, rheumatoid arthritis and inflammatory bowel disease.

Model	APR@5 Top-20 CT	AUROC Top-1 CT	AUROC Top-10 CT	AUROC Top-20 CT	APR@5 CF	AUROC CF

PINNACLE (RA)	0.913±0.059	0.765±0.054	0.676±0.017	0.647±0.014	0.226±0.023	0.510±0.005
GAT (RA)	N/A	N/A	N/A	N/A	0.220±0.013	0.580±0.010

PINNACLE (IBD)	0.873±0.069	0.935±0.067	0.799±0.017	0.752±0.011	0.198±0.013	0.500±0.010
GAT (IBD)	N/A	N/A	N/A	N/A	0.200±0.023	0.640±0.017

Cell-type specific context metrics: APR@5 Top-20 CT - average precision and recall at *k* = 5 for the 20 best-performing cell types (CT); AUROC Top-1 CT - AUROC for top-performing cell type; AUROC Top-10 CT and AUROC Top-20 CT - weighted average AUROC for top-10 and top-20 performing cell types, respectively, each weighted by the number of samples in each cell type; APR@5/AUROC CF - context-free APR@5/AUROC integrated across all cell types. Shown are results from models run on ten independent seeds. N/A - not applicable.

**Table 2:** We’ve evaluated chemCPA utilizing cold splits on perturbation type and show a significant decrease in performance for 3 of 4 perturbations evaluated. We’ve also included Biolord [42] and scGen [92] for comparison. The dataset used was 4 chemical (drug) perturbations from sciPlex2 [2].

Drug	Method	*R*^2^ (seen perturbations)	*R*^2^ (unseen perturbations)

BMS	Baseline	0.620±0.044	N/A
Dex	Baseline	0.603±0.053	N/A
Nutlin	Baseline	0.628±0.036	N/A
SAHA	Baseline	0.617±0.027	N/A

BMS	Biolord	0.939±0.022	N/A
Dex	Biolord	0.942±0.028	N/A
Nutlin	Biolord	0.928±0.026	N/A
SAHA	Biolord	0.980±0.005	N/A

BMS	ChemCPA	0.943±0.006	0.906±0.006
Dex	ChemCPA	0.882±0.014	0.540±0.013
Nutlin	ChemCPA	0.925±0.010	0.835±0.009
SAHA	ChemCPA	0.825±0.026	0.690±0.021

BMS	scGen	0.903±0.030	N/A
Dex	scGen	0.944±0.018	N/A
Nutlin	scGen	0.891±0.032	N/A
SAHA	scGen	0.948±0.034	N/A

**Table 3:** TCR-epitope binding interaction binary classification performance. All models perform poorly under realistic but challenging RN and ET experimental setups. The best-performing model in RN is AVIB-TCR, with an average of 0.576 (AUROC). The best-performing model in ET is MIX-TPI, with an average of 0.700 (AUROC). For NA, 4 of 6 models achieve near-perfect AUROC.

Methods	Experimental setup	ACC	F1	AUROC	AUPRC

AVIB-TCR	RN	0.570±0.028	0.468±0.086	0.576±0.049	0.605±0.044
MIX-TPI	RN	0.539±0.039	0.408±0.122	0.558±0.028	0.597±0.049
Net-TCR2	RN	0.528±0.050	0.354±0.036	0.551±0.042	0.554±0.075
PanPep	RN	0.507±0.028	0.473±0.039	0.535±0.021	0.579±0.040
TEINet	RN	0.459±0.036	0.619±0.036	0.535±0.029	0.581±0.043
TITAN	RN	0.476±0.063	0.338±0.111	0.502±0.066	0.523±0.055

AVIB-TCR	ET	0.611±0.012	0.553±0.020	0.683±0.010	0.815±0.006
MIX-TPI	ET	0.652±0.009	0.523±0.035	0.703±0.016	0.825±0.014
Net-TCR2	ET	0.621±0.027	0.522±0.020	0.674±0.017	0.810±0.016
PanPep	ET	0.556±0.009	0.506±0.011	0.638±0.009	0.753±0.009
TEINet	ET	0.356±0.008	0.512±0.010	0.571±0.009	0.646±0.011
TITAN	ET	0.670±0.013	0.492±0.048	0.624±0.021	0.733±0.018

AVIB-TCR	NA	0.636±0.062	0.197±0.169	0.944±0.021	0.949±0.023
MIX-TPI	NA	0.952±0.029	0.937±0.040	0.992±0.002	0.995±0.001
Net-TCR2	NA	0.655±0.051	0.274±0.123	0.973±0.009	0.985±0.005
PanPep	NA	0.419±0.011	0.352±0.006	0.611±0.014	0.499±0.031
TEINet	NA	0.413±0.023	0.582±0.023	0.973±0.011	0.981±0.006
TITAN	NA	0.695±0.050	0.404±0.141	0.629±0.053	0.661±0.040

**Figure 2:**
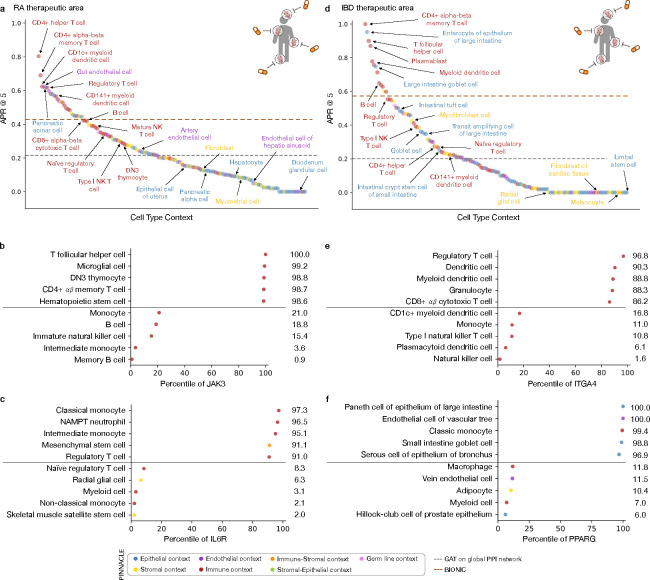
Performance of contextualized target prioritization for RA and IBD therapeutic areas. (a,d) Model performance (measured by APR@5) for RA and IBD therapeutic areas, respectively. APR@K (or Average Precision and Recall at K) is a combination of Precision@K and Recall@K (refer to Methods 6 [3] for more details). Each dot is the performance (averaged across ten random seeds) of PINNACLE’s protein representations from a specific cell type context. The gray and dark orange lines represent the performance of the global reference network model and the BIONIC models, respectively. For each therapeutic area, 22 cell types are annotated and colored by their compartment category. Supplementary Figure S2 contains model performance measured by APR@10, APR@15, and APR@20 for RA and IBD therapeutic areas. (b-c, e-f) Selected proteins for RA and IBD therapeutic areas. The dotted line separates the top and bottom five cell types. (b-c) Two selected proteins, JAK3 and IL6R, are targeted by drugs that have completed Phase IV clinical trials for treating RA therapeutic areas. (e-f) Two selected proteins, ITGA4 and PPARG, are targeted by drugs that have completed Phase IV for treating the therapeutic area of IBD.

**Figure 4:**
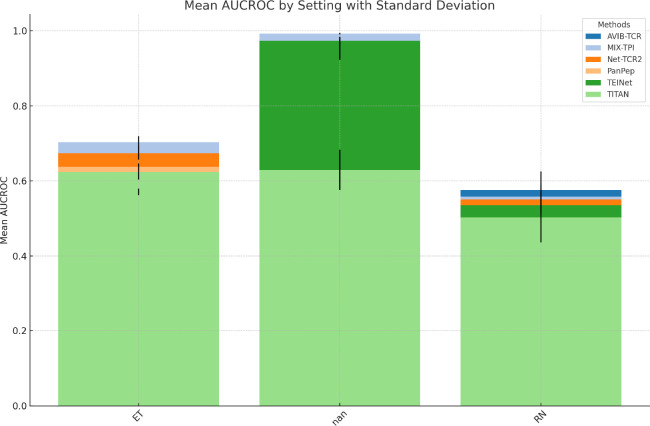
AUROC for TCR-Epitope Binding Interaction Binary Classification model performance across negative sampling methods.

## A Technical Appendix

This technical appendix provides a detailed overview of the design, tasks, and benchmarks introduced by TDC-2. We also refer to materials in Section C, Supplementary Information, throughout the technical appendix.

### A.1 TDC-2 Multimodal Retrieval Use Cases

We focus on the use case of an ML researcher who wishes to train a model on a large-scale single-cell atlas. In particular, researchers would be familiar with and have trained models on traditional single-cell datasets such as Tabula Sapiens [71]. Their interest is to scale a model by training it on a more extensive single-cell atlas based on this reference dataset. We build such an API. Specifically, given a reference dataset available in CellXGene Discover [53], we allow the user to perform a memory-efficient query using TileDB-SOMA to expand the reference dataset to include cell entries with non-zero readouts for any of the genes present in the reference dataset. This allows users to build large-scale single-cell atlases on familiar reference datasets. The example below illustrates how a user may construct a large-scale atlas with Tabula Sapiens as the reference dataset. Other use cases include augmenting datasets using knowledge graphs and cell-type-specific biomedical contexts. These capabilities are all powered by the MVC (Section 3.2) and DSL (Section A.2.1 and Section 3.2).

### A.2 TDC-2 Design and Code Supporting Materials

All code and documentation can be found in our Github repo. The URL is https://github.com/mims-harvard/TDC/tree/main. In addition, our website contains all datasets and licenses and further documentation https://tdcommons.ai/.

#### A.2.1 DSL Implementation Examples

The above configuration augments a protein-peptide dataset with an additional modality, amino acid sequence, and invokes numerous data processing functions tailored to the specific needs of the underlying dataset.

#### A.2.2 CellXGene Code Samples

We focus on the use case of an ML researcher who wishes to train a model on a large-scale single-cell atlas. In particular, researchers would be familiar with and would have trained models on traditional single-cell datasets such as Tabula Sapiens [71]. Their interest is to scale a model by training it on a more extensive single-cell atlas based on this reference dataset. We build such an API. Specifically, given a reference dataset available in CellXGene Discover [53], we allow the user to perform a memory-efficient query using TileDB-SOMA to expand the reference dataset to include cell entries with non-zero readouts for any of the genes present in the reference dataset. This allows users to build large-scale single-cell atlases on familiar reference datasets. The example below illustrates how a user may construct a large-scale atlas with Tabula Sapiens as the reference dataset.

In addition to our TDC-2 DataLoader API implementation for the CellXGene RPC API, we provide a wrapper over the CellXGene Census Discovery API, which allows users to perform remote procedure calls to fetch Cell Census data in more machine-learning-friendly formats like Pandas and Scipy. We also maintain support for the AnnData format. Users can query Cell Census counts as well as metadata using this API. The code sample below illustrates such usage.

#### A.2.3 PrimeKG Knowledge Graph

PrimeKG supports drug-disease prediction by including an abundance of ‘indications,’ ‘contradictions’, and ‘off-label use’ edges, which are usually missing in other knowledge graphs. We accompany PrimeKG’s graph structure with text descriptions of clinical guidelines for drugs and diseases to enable multimodal analyses [63]. The code below depicts an example use case of the TDC-2 PrimeKG API, where, combined with the networkx module, a user may retrieve a set of proteins a drug interacts with.

#### A.2.4 The Commons 2.0’s HuggingFace Model Hub

TDC-2 introduces The Commons’ HuggingFace Model Hub. It is a resource with pre-trained models, including geometric deep learning models, large language models, and other contextualized multimodal models for therapeutic tasks. The models can be fine-tuned using datasets in TDC-2 and be used for downstream tasks such as implementations of multi-agent collaborative schemes [31] (i.e., expert consultants).

### A.3 Task Definitions and Benchmark Results

Here, we provide details of mathematical formulation, definitions, and benchmark results for all new tasks. Complete derivations are available in Section C. Section C also contains complete descriptions for all datasets across these tasks.

#### A.3.1 Contextualized Drug Target Identification Task Formulation

For complete mathematical formulation of the drug-target nomination (identification) task in TDC-2, please see Section C.2.1. Section C.2.1 also contains complete dataset descriptions.

We created a curated dataset for benchmarking models on single-cell drug-target identification by replicating the methodology used for evaluating PINNACLE [3]. We used curated therapeutic target labels from the Open Targets Platform [3, 4] for rheumatoid arthritis (RA) and inflammatory bowel disease (IBD). Positive examples were defined by proteins targeted by drugs that have at least completed phase 2 of clinical trials. The final number of positive (negative) samples for RA and IBD were 152 (1,465) and 114 (1,377), respectively. This dataset was augmented to include 156 cell-type-specific contexts.

Section B.1 and Section B.1 for cell-type-specific metrics evaluated across 10 seeds. For benchmarking across ten seeds and another model benchmark, see Appendix B.1. For pre-training, the best hyperparameters are as follows: the dimension of the nodes’ feature matrix = 1024, dimension of the output layer = 16, lambda = 0.1, learning rate for link prediction task = 0.01, learning rate for protein’s cell type classification task = 0.1, number of attention heads = 8, weight decay rate = 0.00001, dropout rate = 0.6, and normalization layers are layernorm and batchnorm. For pre-training, models are trained on a single NVIDIA Tesla V100-SXM2–16GB GPU.

Hyperparameters are used for fine-tuning, as per the Github documentation. Models are trained on a single NVIDIA Tesla M40 GPU.

The following function calls are as documented in Section B.1.

#### A.3.2 Perturbation-Response Problem Formulation

TDC-2 introduces the Contextualized Perturbation-Response Prediction task. The predictive, non-generative task is formalized as learning an estimator for a function of the cell-type-specific gene expression response to a chemical or genetic perturbation, taking a perturbation p∈P, a pre-perturbation gene expression profile from the control set $e_{0}\in E_{⊬}$, and the biological context c∈C under which the gene expression response to the perturbation is measured as:

(1)
$$\vec{e_{1}}=f(p,\;\vec{e_{0}},\;c).$$

We center our definition on regression for the cell-type-specific gene expression vector in response to a chemical or genetic perturbation.

#### Perturbation set.

The perturbation set includes genetic and chemical perturbations. It is denoted by:

(2)
$$P=\{p_{1},\ldots ,p_{N_{p}}\},$$

where $t_{p},\ldots ,p_{N_{p}}$ are $N_{p}$ evaluated perturbations. Data representation models for genetic perturbations can include the type of perturbation (i.e., knockout, knockdown, overexpression) and target gene(s) of the perturbation. Information modeled for chemical perturbations can include chemical structure (i.e., SMILES, InChl) and concentration and duration of treatment.

##### Control set.

The control set includes the unperturbed gene expression profiles. This set is denoted as:

(3)
$$E_{⊬}=\{{\vec{e}}_{0_{1}},\ldots ,{\vec{e}}_{N_{e_{0}}}\},$$

where ${\vec{e}}_{0_{1}},\ldots ,{\vec{e}}_{N_{e_{0}}}$ are $N_{e_{0}}$ unperturbed gene expression profile vectors. Data representation models for gene expression profiles include raw or normalized gene expression counts, transcriptomic profiles, and isoform-specific expression levels.

##### Biological context set.

The biological context set includes the cell-type-specific contexts under which the perturbed gene expression profile is measured. It is denoted by:

(4)
$$C=\left\{c_{1},\ldots ,c_{N_{c}}\right\},$$

where $c_{1},\ldots ,c_{N_{c}}$ are the $N_{c}$ biological contexts under which perturbations are being evaluated. Information modeled for biological contexts can include cell type or tissue type and experimental conditions [2] as well as epigenetic markers [122, 123].

##### Perturbation-response readouts.

Perturbation-Response is a gene expression vector $\vec{e_{1}}$, where $\vec{e_{1i}}$ denotes the expression of the i-th gene in the vector. It is the outcome of applying a perturbation, $p_{i}\in P$, within a biological context, $c_{j}\in C$, to a cell with a measured control gene expression vector, $e_{0_{k}}\in E_{⊬}$.

The Perturbation-Response Prediction learning task is to learn a regression model $f_{\theta }$ estimating the perturbation-response gene expression vector $\overset{ˆ}{\vec{e_{1}}}$ for a perturbation applied in a cell-type-specific biological context to a control:

(5)
$$\overset{ˆ}{\overset{\to }{e_{1}}}=f_{\theta }(p\in P,\;\overset{\to }{e_{0}}\in E_{⊬},\;c\in C).$$

##### Benchmarking genetic perturbations.

All benchmarked methods follow the training procedure described in [19]. Specifically, we use the simulation data split to mimic the real-world use case of genetic perturbation machine learning models. For the Norman double combination perturbation dataset, we withhold perturbations that are either both unseen, one unseen, or both seen in the test set. For the Replogle K562 and RPE1 single perturbation datasets, we split the data by single genes and test on unseen single-gene perturbations. The hyperparameters used were optimal after optimization as reported in [19]. Each model run was executed on an internal high-performance cluster with an Ubuntu 16.04 operating system, using a single Nvidia Quadro RTX 8000 48GB GPU. The code to reproduce the experiment is available at https://github.com/mims-harvard/TDC/tree/main/examples/multi_pred/geneperturb and results at https://docs.google.com/spreadsheets/d/1ZRONBsJasCgtytz_btId0RRmIwbMmtGGlrWyDMqBKwM/edit?usp=sharing.

##### Benchmarking chemical perturbations.

Benchmark results can be reproduced with code in B.1. Default settings were used from each model’s github repo and the experiments were run in A100. Below is code chemical and genetic perturbation benchmarking tooling available in TDC-2. https://tdcommons.ai/benchmark/counterfactual_group/overview/ https://github.com/mims-harvard/TDC/blob/main/tdc/benchmark_group/counterfactual_group.py https://github.com/mims-harvard/TDC/blob/main/tdc/benchmark_group/geneperturb_group.py

#### A.3.3 Protein-Peptide Interaction Prediction Problem Formulation

TDC-2 introduces the Protein-Peptide Binding Affinity Prediction task. The predictive, non-generative task is to learn a model estimating a function of a protein, peptide, antigen processing pathway, biological context, and interaction features. It outputs a binding affinity value (e.g., dissociation constant Kd, Gibbs free energy ΔG) or binary label indicating strong or weak binding. The binary label can also include additional biomarkers, such as allowing for a positive label if and only if the binding interaction is specific [9, 124, 125]. Our task is specified with a binary label to account for additional biomarkers beyond binding affinity value.

##### Protein set.

The protein set includes target proteins. It is denoted by:

(6)
$$P=\{p_{1},\ldots ,p_{N_{p}}\},$$

where $p_{1},\ldots ,p_{N_{p}}$ are $N_{p}$ target proteins. Information modeled for proteins can include sequence, structural, or post-translational modification data.

##### Peptide set.

The control set includes the peptide candidates. This set is denoted as:

(7)
$$S=\left\{s_{1},\ldots ,s_{N_{s}}\right\},$$

where $s_{1},\ldots ,s_{N_{s}}$ are $N_{s}$ candidate peptides. Information modeled for candidate peptides can include sequence, structural, and physicochemical data.

##### Antigen processing pathway set.

The antigen processing pathway set includes antigen processing pathway profile information about prior steps in the biological antigen presentation pathway processes. It is denoted by:

(8)
$$A=\left\{a_{1},\ldots ,a_{N_{a}}\right\},$$

where $a_{1},\ldots ,a_{N_{a}}$ are the $N_{a}$ antigen processing pathway profiles modeled. Information modeled in a profile can include proteasomal cleavage sites [126], classification into viral, bacterial, and self-protein sources and endogenous vs exogenous processing pathway [99, 127, 110, 128], and target/receptor-specific pathway attributes such as transporter associated with antigen processing (TAP) affinity [129], and endosomal/lysosomal processing efficiency [130].

##### Interaction set.

It contains the interaction feature profiles. The set is denoted by:

(9)
$$I=\left\{i_{1},\ldots ,i_{N_{i}}\right\},$$

where $i_{1},\ldots ,i_{N_{i}}$ are the $N_{i}$ interaction feature profiles. Information modeled in an interaction feature profile can include contact maps [131, 97, 132, 133], distance maps [97, 134], electrostatic interactions [131], and hydrogen bonds [131].

##### Cell-type-specific biological context set.

It contains the interaction feature profiles. The set is denoted by:

(10)
$$C=\left\{c_{1},\ldots ,c_{N_{c}}\right\},$$

where $c_{1},\ldots ,c_{N_{c}}$ are the $N_{c}$ cell-type-specific biological contexts under which the protein-peptide interaction is being evaluated. Information modeled in the cell-type-specific biological context can include transcriptomic and proteomic data. We note, however, that, to our knowledge, single-cell transcriptomic and proteomic data has yet to be used in protein-peptide binding affinity prediction, outlining a promising avenue of research in developing machine learning models for peptide-based therapeutics.

##### Protein-peptide interaction.

It is defined as a binary label, y∈{1,0}, where y=1 indicates a protein-peptide pair met the target biomarkers and y=0 indicates the pair did not meet the target biomarkers.

The Protein-Peptide Interaction Prediction learning task is to learn a binary classification model $f_{\theta }$ estimating the probability, $\overset{ˆ}{y}$, of a protein-peptide interaction meeting specific biomarkers:

(11)
$$\overset{ˆ}{y}=f_{\theta }\left(p\in P,\;s\in S,\;a\in A,\;i\in I,c\in C\right).$$

The models for TCR-epitope binding prediction were run on a single A100. We prepared the input data files in the format (most in CSV files) according to the official tutorials. Unknown amino acid letters were replaced by X or removed according to the method requirements. If CDR3A and CDR3B are available, the models will be trained on both unless they can only accept one TCR sequence as input (such as TITAN). If CDR3A is unavailable (ET data), all the models will be trained in the beta-only module. We kept the default parameters to run all the methods. For running TITAN, we transferred the amino acid sequences of epitopes to the SMILE sequences as the inputs. To keep the unseen scenario, we used a zero-shot module of PanPep in the tests of all the data settings. The code for reproducing our benchmark results is in Section B.1. The code for our data split and benchmarks tooling is available at. https://github.com/mims-harvard/TDC/blob/main/tdc/benchmark_group/tcrepitope_group.py https://tdcommons.ai/benchmark/proteinpeptide_group/overview/https://tdcommons.ai/multi_pred_tasks/tcrepitope/

###### AS-MS Data for newly discovered ligands - Protein-Peptide Binding Affinity Prediction

To benchmark future generalized protein-peptide models for this task, we use affinity selection-mass spectrometry data which identified ligands binding to single biomolecular targets (MDM2, ACE2, 12ca5) [119, 9]. This dataset contains affinity selection-mass spectrometry data of discovered ligands against single biomolecular targets. Several ligands identified through AS-MS were further tested for binding affinity (KD) using biolayer interferometry (BLI) to the listed target protein. If labeled as a “putative binder,” AS-MS alone was used to isolate the ligands, with a requirement of KD < 1 uM, often confirmed in other assays but with some (< 50%) chance of nonspecific binding. Most of the ligands are putative binders, totaling 4446. Among those characterized by BLI (only 34), the average KD is 266 ± 44 nM, and the median KD is 9.4 nM. We anticipate this new dataset will help bridge the gap between novel experimental chemistry results and computational protein-peptide binding affinity prediction, aiding in establishing model generalizability for benchmarks. A limitation of the standalone dataset is the lack of several modalities mentioned in the problem formulation. Furthermore, this dataset will be augmented using the TDC-2 MVC.

**Table 4:** SOTA TCR-Epitope Binding Interaction Binary Classification model AUROC performance across negative sampling methods.

Model	NA AUROC	ET AUROC	RN AUROC

Titan	0.629	0.624	0.481
AVIB-TCR	0.962	0.683	0.567
MIX-TPI	0.989	0.703	0.559
TEINET	0.973	0.571	0.609
NetTCR2	0.991	0.674	0.552
PanPep	0.611	0.674	0.535

**Table 5:** TCR-Epitope Binding Interaction Binary Classification model performance across negative sampling methods.

Methods	Setting	ACC	F1	AUROC	AUPRC

AVIB-TCR	ET	0.611±0.012	0.553±0.020	0.683±0.010	0.815±0.006
MIX-TPI	ET	0.652±0.009	0.523±0.035	0.703±0.016	0.825±0.014
Net-TCR2	ET	0.621±0.027	0.522±0.020	0.674±0.017	0.810±0.016
PanPep	ET	0.556±0.009	0.506±0.011	0.638±0.009	0.753±0.009
TEINet	ET	0.356±0.008	0.512±0.010	0.571±0.009	0.646±0.011
TITAN	ET	0.670±0.013	0.492±0.048	0.624±0.021	0.733±0.018

AVIB-TCR	NA	0.636±0.062	0.197±0.169	0.944±0.021	0.949±0.023
MIX-TPI	NA	0.952±0.029	0.937±0.040	0.992±0.002	0.995±0.001
Net-TCR2	NA	0.655±0.051	0.274±0.123	0.973±0.009	0.985±0.005
PanPep	NA	0.419±0.011	0.352±0.006	0.611±0.014	0.499±0.031
TEINet	NA	0.413±0.023	0.582±0.023	0.973±0.011	0.981±0.006
TITAN	NA	0.695±0.050	0.404±0.141	0.629±0.053	0.661±0.040

AVIB-TCR	RN	0.570±0.028	0.468±0.086	0.576±0.049	0.605±0.044
MIX-TPI	RN	0.539±0.039	0.408±0.122	0.558±0.028	0.597±0.049
Net-TCR2	RN	0.528±0.050	0.354±0.036	0.551±0.042	0.554±0.075
PanPep	RN	0.507±0.028	0.473±0.039	0.535±0.021	0.579±0.040
TEINet	RN	0.459±0.036	0.619±0.036	0.535±0.029	0.581±0.043
TITAN	RN	0.476±0.063	0.338±0.111	0.502±0.066	0.523±0.055

#### A.3.4 Clinical Trial Outcome Prediction Problem Formulation

The Clinical Trial Outcome Prediction task is formulated as a binary classification problem, where the machine learning model predicts whether a clinical trial will have a positive or negative outcome. It is a function that takes patient data, trial design, treatment characteristics, disease, and macro variables as inputs and outputs a trial outcome prediction, a binary indicator of trial success (1) or failure (0).

##### Patient set.

The patient set includes one or multiple patient sub-populations, with the extreme case representing personalization. It is denoted as follows:

(12)
$$P=\{p_{1},\ldots ,p_{N_{p}}\},$$

where $p_{1},\ldots ,p_{N_{p}}$ are $N_{p}$ patient sub-populations in this trial. The TOP benchmark [8] dataset represents patient data as part of the trial eligibility criteria. Patient data can include demographics [135, 136, 137, 138, 139], baseline health metrics [138, 139, 140], and medical history [135, 136, 137, 138, 139].

##### Trial design set.

The trial design set includes the trial design profiles for this clinical trial. It is denoted as:

(13)
$$D=\left\{d_{1},\ldots ,d_{N_{d}}\right\},$$

where $d_{1},\ldots ,d_{N_{d}}$ are $N_{d}$ eligible trial design profiles for this clinical trial. Trial design profiles can model information including phase of the trial [8], number of participants, duration of the trial, trial eligibility criteria [8], and randomization and blinding methods [141, 142, 143].

##### Treatment set.

The treatment set includes the candidate treatments for the trial. It is denoted as:

(14)
$$T=\left\{t_{1},\ldots ,t_{N_{t}}\right\},$$

where $t_{1},\ldots ,t_{N_{t}}$ are $N_{t}$ candidate treatments for the clinical trial. The information modeled for treatments can include type of treatment (drug [8, 144], device [145, 146, 147], procedure [148, 149, 150, 151, 152]), dosage and administration route [141, 140, 153], mechanism of action [154, 155, 156], pre-clinical and early-phase trial results [155, 140, 157, 158].

##### Macro context set.

The macro context set contains the configurations of macro variables relevant to the clinical trial. It is denoted as:

(15)
$$C=\left\{c_{1},\ldots ,c_{N_{c}}\right\},$$

where $c_{1},\ldots ,c_{N_{c}}$ are $N_{c}$ configurations containing the values for macro variables relevant to the trial, which can include geography [159, 155, 158, 160] and regulatory considerations [155, 159].

##### Trial outcome.

The trial outcome is a binary label y∈{1,0}, where y=1 indicates the trial met their primary endpoints, while 0 means failing to meet with the primary endpoints.

The learning task is to learn a model $f_{\theta }$ f for predicting the trial success probability probability $\overset{ˆ}{y}$, where $\overset{ˆ}{y}\in [0,\;1]$:

(16)
$$\overset{ˆ}{y}=f_{\theta }\left(p\in P,\;d\in D,\;t\in T,\;c\in C\right).$$

#### A.3.5 Dataset and Benchmark

Our benchmark uses the Trial Outcome Prediction (TOP) dataset [8]. TOP consists of 17,538 clinical trials with 13,880 small-molecule drugs and 5,335 diseases. Out of these trials, 9,999 (57.0%) succeeded (i.e., meeting primary endpoints), and 7,539 (43.0%) failed. Out of these trials, 1,787 were in Phase I testing (toxicity and side effects), 6,102 in Phase II (efficacy), and 4,576 in Phase III (effectiveness compared to current standards). We perform a temporal split for benchmarking. The train/validation and test are time-split by the date January 1, 2014, i.e., the start dates of the test set are after January 1, 2014, while the completion dates of the train/validation set are before January 1, 2014. Here, we cite the HINT model [8], which is benchmarked against COMPOSE [161] and DeepEnroll [162] models.

**Table 6:** Clinical Trial Outcome Prediction task benchmark model results on the TOP dataset [8].

Model	Phase 1 AUPRC	Phase 2 AUPRC	Phase 3 AUPRC	Indication Level AUPRC

HINT	0.772	0.607	0.623	0.703
COMPOSE	0.665	0.532	0.545	0.624
DeepEnroll	0.701	0.580	0.590	0.655

TDC-2 augments this task with additional datasets and benchmarks for the modalities discussed in the problem formulation. The benchmark can be reproduced using the following https://github.com/futianfan/clinical-trial-outcome-prediction.

### A.4 Structure-Based Drug Design Task

Complete task formulation, definitions, and dataset descriptions are available in Section C.2.5. You may additionally see https://tdcommons.ai/generation_tasks/sbdd/ for tasks and datasets.

## B Additional Materials

Here, we include external support material. This mainly consists of links to external code and data sources used for benchmarking and website links.

### B.1 Model Benchmarking

TDC-2 Benchmarking Tooling Code for Chemical Perturbations https://github.com/mims-harvard/TDC/blob/main/tdc/benchmark_group/counterfactual_group.py

TDC-2 Benchmarking Tooling Code for CRISPR-based Perturbations https://github.com/mims-harvard/TDC/blob/main/tdc/benchmark_group/geneperturb_group.py

Reproducing Benchmark Results for Clinical Trial Outcome Prediction https://github.com/futianfan/clinical-trial-outcome-prediction

Evaluating Cell-Type-Specific Context Metrics for PINNACLE Across 10 Seeds https://colab.research.google.com/drive/1gjZIfmF2Gmz3Nqm1uGP79l0AmsPAvj_5?usp=sharing

Evaluating Cell-Type-Specific Context Metrics for PINNACLE Across 10 Seeds. Outputs Referenced in PINNACLE [3] and its reproducibility documentation https://drive.google.com/drive/folders/1QX05afMekucbtjl_07ZxZhgnKVH3OXMk?usp=sharing

Code for Benchmarking PINNACLE. Additional Details are in [3] https://github.com/mims-harvard/PINNACLE/tree/main/evaluate

Reproducing TCR-Epitope results. Code for Benchmarking models in 4.3.1. A bash script for each negative sampling method is included for each TCR-Epitope model. Instructions are in A.3.3. https://drive.google.com/drive/folders/1O7G_h_06VDABM6U_Xt7otXPazK0XTAG9?usp=sharing

Reproducing Chemical Perturbation results. Code for Benchmarking models in 4.2 chemical perturbation section. A run_chemical_sc.py Python script is included for each model. Default settings were used from each model’s GitHub repo, and the experiments were run in A100. https://drive.google.com/drive/folders/1RlBnRPmWFRQ6M_1EQ_FMwFb1Y8IjXoyC?usp=sharing

### B.2 Leaderboards

TDC.PerturbOutcome Leaderboard https://tdcommons.ai/benchmark/counterfactual_group/overview/

TDC.ProteinPeptide Leaderboard https://tdcommons.ai/benchmark/proteinpeptide_group/overview/

TDC.scDTI Leaderboard https://tdcommons.ai/benchmark/scdti_group/overview/

## C Supplementary Information

### C.1 TDC-2 Codebase and Documentation

All code and documentation for TDC-2 are available in our Github repo https://github.com/mims-harvard/TDC. The Therapeutic Data Commons website includes further information on the project, team members, datasets, data processing functions, TDC-1 publications, and the Model Hub. The website can be reached at https://tdcommons.ai/. The TDC-2 Model Hub is available at https://huggingface.co/tdc.

### C.2 Task Definitions and Datasets

#### C.2.1 TDC.scDTI: Contextualized Drug-Target Nomination (Identification)

TDC-2 introduces TDC.scDTI task. The predictive, non-generative task is formalized as learning an estimator for a function f of a target protein and cell type outputting whether the candidate protein t is a therapeutic target in that cell type c:

(17)
$$y=f\left(t,\;c\right).$$

##### Target candidate set.

The target candidate set includes proteins, nucleic acids, or other molecules drugs can interact with, producing a therapeutic effect or causing a biological response. The target candidate set is constrained to proteins relevant to the disease being treated. It is denoted by:

(18)
$$T=\left\{t_{1},\ldots ,t_{N_{t}}\right\},$$

where $t_{1},\ldots ,t_{N_{t}}$ are $N_{t}$ target candidates for the drugs treating the disease. Information modeled for target candidates can include interaction, structural, and sequence information.

##### Biological context set.

The biological context set includes the cell-type-specific contexts in which the target candidate set operates. This set is denoted as:

(19)
$$C=\left\{c_{1},\ldots ,c_{N_{c}}\right\},$$

where $c_{1},\ldots ,c_{N_{c}}$ are $N_{c}$ biological contexts on which drug-target interactions are being evaluated. Information modeled for cell-type-specific biological contexts can include gene expression and tissue hierarchy. The set is constrained to disease-specific cell types and tissues.

##### Drug-target identification.

Drug-Target Identification is a binary label y∈{1,0}, where y=1 indicates the protein is a candidate therapeutic target. At the same time, 0 means the protein is not such a target.

The goal is to train a model $f_{\theta }$ for predicting the probability $\overset{ˆ}{y}\in [0,\;1]$ that a protein is a candidate therapeutic target in a specific cell type. The model learns an estimator for a function of a protein target t∈T and a cell-type-specific biological context c∈C as input, and the model is tasked to predict:

(20)
$$\overset{ˆ}{y}=f_{\theta }\left(t\in T,c\in C\right).$$

##### (Li, Michelle, et al.) Dataset

To curate target information for a therapeutic area, we examine the drugs indicated for the therapeutic area of interest and its descendants. The two therapeutic areas examined are rheumatoid arthritis (RA) and inflammatory bowel disease. For rheumatoid arthritis, we collected therapeutic data (i.e., targets of drugs indicated for the therapeutic area) from OpenTargets for rheumatoid arthritis (EFO 0000685), ankylosing spondylitis (EFO 0003898), and psoriatic arthritis (EFO 0003778). For inflammatory bowel disease, we collected therapeutic data for ulcerative colitis (EFO 0000729), collagenous colitis (EFO 1001293), colitis (EFO 0003872), proctitis (EFO 0005628), Crohn’s colitis (EFO 0005622), lymphocytic colitis (EFO 1001294), Crohn’s disease (EFO 0000384), microscopic colitis (EFO 1001295), inflammatory bowel disease (EFO 0003767), appendicitis (EFO 0007149), ulcerative proctosigmoiditis (EFO 1001223), and small bowel Crohn’s disease (EFO 0005629).

We define positive examples (i.e., where the label y = 1) as proteins targeted by drugs that have at least completed phase 2 of clinical trials for treating a specific therapeutic area. As such, a protein is a promising candidate if a compound that targets the protein is safe for humans and effective for treating the disease. We retain positive training examples activated in at least one cell type-specific protein interaction network.

We define negative examples (i.e., where the label y = 0) as druggable proteins that do not have any known association with the therapeutic area of interest according to Open Targets. A protein is deemed druggable if targeted by at least one existing drug. We extract drugs and their nominal targets from Drugbank. We retain negative training examples activated in at least one cell type-specific protein interaction network.

##### Dataset statistics.

The final number of positive (negative) samples for RA and IBD were 152 (1,465) and 114 (1,377), respectively. In [3], this dataset was augmented to include 156 cell types.

##### Dataset split. Cold Split:

We split the dataset such that about 80% of the proteins are in the training set, about 10% of the proteins are in the validation set, and about 10% of the proteins are in the test set. The data splits are consistent for each cell type context to avoid data leakage.

References. [3]

Dataset license. CC BY 4.0

##### Code Sample

The dataset and splits are currently available on TDC Harvard Dataverse. https://dataverse.harvard.edu/file.xhtml?fileId=10143574&version=86.0 https://dataverse.harvard.edu/file.xhtml?fileId=10143573&version=86.0 In addition, you may obtain the protein splits used in [3] via the following code.

##### Contextualized Drug-Target Interaction

###### Contextualized drug target interaction task.

We formalize the predictive, non-generative task definition as learning an estimator for a function of the chemical association between the drug and target, taking biomolecules from the target set t∈T, their cell-type-specific biological context c∈C, and a drug from the candidate set d∈D as input:

(21)
$$y=f\left(t,\;c,\;d\right).$$

We center our formulation around the cell-type-specific context in which a target operates and binary classification on the drug-target interaction of interest, such as whether the protein and drug will bind with strong affinity.

###### Target candidate set.

The target candidate set includes proteins, nucleic acids, or other molecules drugs can interact with, producing a therapeutic effect or causing a biological response. It is denoted by:

(22)
$$T=\left\{t_{1},\ldots ,t_{N_{t}}\right\},$$

where $t_{1},\ldots ,t_{N_{t}}$ are $N_{t}$ target candidates for the evaluated set of drugs. Data representation models for target candidates can include interaction, structural, and sequence information.

###### Biological context set.

The biological context set includes the cell-type-specific contexts in which the target candidate set operates. This set is denoted as:

(23)
$$C=\left\{c_{1},\ldots ,c_{N_{c}}\right\},$$

where $c_{1},\ldots ,c_{N_{c}}$ are $N_{c}$ biological contexts on which drug-target interactions are being evaluated. Data representation models for cell-type-specific biological contexts can include gene expression and tissue hierarchy. The set can be constrained to the most relevant contexts, such as disease or perturbation-specific cell types and tissues.

###### Drug candidate set.

The drug candidate set includes the drug molecules tested for a particular therapeutic effect or biological response. It is denoted by:

(24)
$$D=\left\{d_{1},\ldots ,d_{N_{d}}\right\},$$

where $d_{1},\ldots ,d_{N_{d}}$ are the $N_{d}$ drug molecules being evaluated. Drug modeling can include molecular structure, often represented in formats such as SMILES (Simplified Molecular Input Line Entry System) or InChI (International Chemical Identifier) [163], physicochemical properties like hydrophobicity and molecular weight [105], and molecular descriptors and fingerprints [164].

###### Drug-target interaction.

Drug-target interaction is a binary label y∈{1,0}, where y=1 indicates the drug-target interaction met its primary biomarker endpoints. At the same time, 0 means failing to meet the primary biomarker endpoints.

The learning task is to learn a model $f_{\theta }$ for predicting the probability $\overset{ˆ}{y}$, where $\overset{ˆ}{y}\in [0,\;1]$, of a drug-target pair meeting the primary biomarker endpoints while interacting in a cell-type-specific biological context:

(25)
$$\overset{ˆ}{y}=f_{\theta }\left(t\in T,\;d\in D,\;c\in C\right).$$

#### C.2.2 TDC.PerturbOutcome: Perturbation-Response Problem Formulation

TDC-2 introduces Perturbation-Response prediction task. The predictive, non-generative task is formalized as learning an estimator for a function of the cell-type-specific gene expression response to a chemical or genetic perturbation, taking a perturbation p∈P, a pre-perturbation gene expression profile from the control set $e_{0}\in E_{⊬}$, and the biological context c∈C under which the gene expression response to the perturbation is being measured:

(26)
$$y=f\left(p,\;e_{0},\;c\right).$$

We center our definition on regression for the cell-type-specific gene expression vector in response to a chemical or genetic perturbation.

#### Perturbation set.

The perturbation set includes genetic and chemical perturbations. It is denoted by:

(27)
$$P=\{p_{1},\ldots ,p_{N_{p}}\},$$

where $t_{p},\ldots ,p_{N_{p}}$ are $N_{p}$ evaluated perturbations. Information modeled for genetic perturbations can include the type of perturbation (i.e., knockout, knockdown, overexpression) and target gene(s) of the perturbation. Information modeled for chemical perturbations can include chemical structure (i.e., SMILES, InChl) and concentration and duration of treatment.

##### Control set.

The control set includes the unperturbed gene expression profiles. This set is denoted as:

(28)
$$E_{⊬}=\{{\overset{\to }{e}}_{0_{1}},\ldots ,{\overset{\to }{e}}_{N_{e_{0}}}\},$$

where ${\overset{\to }{e}}_{0_{1}},\ldots ,{\overset{\to }{e}}_{N_{e_{0}}}$ are $N_{e_{0}}$ unperturbed gene expression profile vectors. Information models for gene expression profiles can include raw or normalized gene expression counts, transcriptomic profiles, and isoform-specific expression levels.

##### Biological context set.

The biological context set includes the cell-type-specific contexts under which the perturbed gene expression profile is measured. It is denoted by:

(29)
$$C=\left\{c_{1},\ldots ,c_{N_{c}}\right\},$$

where $c_{1},\ldots ,c_{N_{c}}$ are the $N_{c}$ biological contexts under which perturbations are being evaluated. Information modeled for biological contexts can include cell type or tissue type and experimental conditions [2] as well as epigenetic markers [122, 123].

##### Perturbation-response readouts.

Perturbation-Response is a gene expression vector $\overset{\to }{e_{1}}$, where $\overset{\to }{e_{1i}}$ denotes the expression of the i-th gene in the vector. It is the outcome of applying a perturbation, $p_{i}\in P$, within a biological context, $c_{j}\in C$, to a cell with a measured control gene expression vector, $e_{0_{k}}\in E_{⊬}$.

The Perturbation-Response Prediction learning task is to learn a regression model $f_{\theta }$ estimating the perturbation-response gene expression vector $\overset{ˆ}{\vec{e_{1}}}$ for a perturbation applied in a cell-type-specific biological context to a control:

(30)
$$\overset{ˆ}{\overset{\to }{e_{1}}}=f_{\theta }\left(p\in P,e_{0}\in E_{⊬},c\in C\right).$$

##### scPerturb Dataset

The scPerturb dataset is a comprehensive collection of single-cell perturbation data harmonized to facilitate the development and benchmarking of computational methods in systems biology. It includes various types of molecular readouts, such as transcriptomics, proteomics, and epigenomics. scPerturb is a harmonized dataset that compiles single-cell perturbation-response data. This dataset is designed to support the development and validation of computational tools by providing a consistent and comprehensive resource. The data includes responses to various genetic and chemical perturbations, crucial for understanding cellular mechanisms and developing therapeutic strategies. Data from different sources are uniformly pre-processed to ensure consistency. Rigorous quality control measures are applied to maintain high data quality. Features across different datasets are standardized for easy comparison and integration.

##### Dataset statistics.

44 publicly available single-cell perturbation-response datasets. Most datasets have, on average, approximately 3000 genes measured per cell. 100,000+ perturbations.

Dataset split.

**Cold Split** and **Random Split** defined on cell lines and perturbation types.

References. [2]

Dataset license. CC BY 4.0

#### C.2.3 TDC.ProteinPeptide: Protein-Peptide Interaction Prediction Problem Formulation

TDC-2 introduces the Protein-Peptide Binding Affinity prediction task. The predictive, non-generative task is to learn a model estimating a function of a protein, peptide, antigen processing pathway, biological context, and interaction features. It outputs a binding affinity value (e.g., dissociation constant Kd, Gibbs free energy ΔG) or binary label indicating strong or weak binding. The binary label can also include additional biomarkers, such as allowing for a positive label if and only if the binding interaction is specific [9, 124, 125]. To account for additional biomarkers beyond binding affinity value, our task is specified with a binary label.

##### Protein set.

The protein set includes target proteins. It is denoted by:

(31)
$$P=\{p_{1},\ldots ,p_{N_{p}}\},$$

where $p_{1},\ldots ,p_{N_{p}}$ are $N_{p}$ target proteins. Information modeled for proteins can include sequence, structural, or post-translational modification data.

##### Peptide set.

The control set includes the peptide candidates. This set is denoted as:

(32)
$$S=\left\{s_{1},\ldots ,s_{N_{s}}\right\},$$

where $s_{1},\ldots ,s_{N_{s}}$ are $N_{s}$ candidate peptides. Information modeled for candidate peptides can include sequence, structural, and physicochemical data.

##### Antigen processing pathway set.

The antigen processing pathway set includes antigen processing pathway profile information about prior steps in the biological antigen presentation pathway processes. It is denoted by:

(33)
$$A=\left\{a_{1},\ldots ,a_{N_{a}}\right\},$$

where $a_{1},\ldots ,a_{N_{a}}$ are the $N_{a}$ antigen processing pathway profiles modeled. Information modeled in a profile can include proteasomal cleavage sites [126], classification into viral, bacterial, and self-protein sources and endogenous vs exogenous processing pathway [99, 127, 110, 128], and target/receptor-specific pathway attributes such as transporter associated with antigen processing (TAP) affinity [129], and endosomal/lysosomal processing efficiency [130].

##### Interaction set.

It contains the interaction feature profiles. The set is denoted by:

(34)
$$I=\left\{i_{1},\ldots ,i_{N_{i}}\right\},$$

where $i_{1},\ldots ,i_{N_{i}}$ are the $N_{i}$ interaction feature profiles. Information modeled in an interaction feature profile can include contact maps [131, 97, 132, 133], distance maps [97, 134], electrostatic interactions [131], and hydrogen bonds [131].

##### Cell-type-specific biological context set.

It contains the interaction feature profiles. The set is denoted by:

(35)
$$C=\left\{c_{1},\ldots ,c_{N_{c}}\right\},$$

where $c_{1},\ldots ,c_{N_{c}}$ are the $N_{c}$ cell-type-specific biological contexts under which the protein-peptide interaction is being evaluated. Information modeled in the cell-type-specific biological context can include transcriptomic and proteomic data. We note, however, that, to our knowledge, single-cell transcriptomic and proteomic data has yet to be used in protein-peptide binding affinity prediction, outlining a promising avenue of research in developing machine learning models for peptide-based therapeutics.

##### Protein-peptide interaction.

It is a binary label, y∈{1,0}, where y=11 indicates a protein-peptide pair met the target biomarkers and y=0 indicates the pair did not meet the target biomarkers.

The Protein-Peptide Interaction Prediction learning task is to learn a binary classification model $f_{\theta }$ estimating the probability, $\overset{ˆ}{y}$, of a protein-peptide interaction meeting specific biomarkers:

(36)
$$\overset{ˆ}{y}=f_{\theta }\left(p\in P,s\in S,a\in A,i\in I,c\in C\right).$$

##### TCHard Dataset

The TChard dataset is designed for TCR-peptide/-pMHC binding prediction. It includes over 500,000 samples from sources such as IEDB, VDJdb, McPAS-TCR, and the NetTCR-2.0 repository. The dataset is utilized to investigate how state-of-the-art deep learning models generalize to unseen peptides, ensuring that test samples include peptides not found in the training set. This approach highlights the challenges modern deep learning methods face in robustly predicting TCR recognition of peptides not previously encountered in training data.

###### Dataset statistics.

500,000 samples

###### Dataset split.

Cold Split referred to as “Hard” split in [7].

References. [7]

Dataset license.

Non-commercial

##### PanPep Dataset

PanPep is a framework constructed in three levels for predicting the peptide and TCR binding recognition. We have provided the trained meta learner and external memory, and users can choose different settings based on their data available scenarios: Few known TCRs for a peptide: few-shot setting; No known TCRs for a peptide: zero-shot setting; plenty of known TCRs for a peptide: majority setting. More information is available in the Github repo https://github.com/bm2-lab/PanPep.

###### Dataset statistics.

Data from multiple studies involving millions of TCR sequences.

Dataset split.

**Cold Split** referred to as “Hard” split in [6].

References. [6]

Dataset license.

GPL-3.0

##### (Ye X et al) Dataset

Affinity selection-mass spectrometry data of discovered ligands against single biomolecular targets (MDM2, ACE2, 12ca5) from the Pentelute Lab of MIT This dataset contains affinity selection-mass spectrometry data of discovered ligands against single biomolecular targets. Several AS-MS-discovered ligands were taken forward for experimental validation to determine the binding affinity (KD) as measured by biolayer interferometry (BLI) to the listed target protein. If listed as a “putative binder,” AS-MS alone was used to isolate the ligands to the target, with KD < 1 uM required and often observed in orthogonal assays, though there is some (< 50%) chance that the ligand is nonspecific. Most of the ligands are putative binders, with 4446 total provided. For those characterized by BLI (only 34 total), the average KD is 266 ± 44 nM; the median KD is 9.4 nM.

###### Dataset statistics.

34 positive ligands, 4446 putative binders, and three proteins

###### **Dataset Split. Stratified Split** and **N/A Split**:

We provide stratified 10/90 split on train/test as well as “test set only” split.

**References.** [119, 9]

Dataset license.

CC BY 4.0

#### C.2.4 Clinical Trial Outcome Prediction Problem Formulation

The Clinical Trial Outcome Prediction task is formulated as a binary classification problem, where the machine learning model predicts whether a clinical trial will have a positive or negative outcome. It is a function that takes patient data, trial design, treatment characteristics, disease, and macro variables as inputs and outputs a trial outcome prediction, a binary indicator of trial success (1) or failure (0).

##### Patient set.

The patient set includes one or multiple patient sub-populations, with the extreme case representing personalization. It is denoted as follows:

(37)
$$P=\{p_{1},\ldots ,p_{N_{p}}\},$$

where $p_{1},\ldots ,p_{N_{p}}$ are $N_{p}$ patient sub-populations in this trial. The TOP benchmark [8] dataset represents patient data as part of the trial eligibility criteria. Patient data can include demographics [135, 136, 137, 138, 139], baseline health metrics [138, 139, 140], and medical history [135, 136, 137, 138, 139].

##### Trial design set.

The trial design set includes this clinical trial’s design profiles. It is denoted as:

(38)
$$D=\left\{d_{1},\ldots ,d_{N_{d}}\right\},$$

where $d_{1},\ldots ,d_{N_{d}}$ are $N_{d}$ eligible trial design profiles for this clinical trial. Trial design profiles can model information including phase of the trial [8], number of participants, duration of the trial, trial eligibility criteria [8], and randomization and blinding methods [141, 142, 143].

###### Treatment set.

The treatment set includes the candidate treatments for the trial. It is denoted as:

(39)
$$T=\left\{t_{1},\ldots ,t_{N_{t}}\right\},$$

where $t_{1},\ldots ,t_{N_{t}}$ are $N_{t}$ candidate treatments for the clinical trial. The information modeled for treatments can include type of treatment (drug [8, 144], device [145, 146, 147], procedure [148, 149, 150, 151, 152]), dosage and administration route [141, 140, 153], mechanism of action [154, 155, 156], pre-clinical and early-phase trial results [155, 140, 157, 158].

###### Macro context set.

The macro context set contains the configurations of macro variables relevant to the clinical trial. It is denoted as:

(40)
$$C=\left\{c_{1},\ldots ,c_{N_{c}}\right\},$$

where $c_{1},\ldots ,c_{N_{c}}$ are $N_{c}$ configurations containing the values for macro variables relevant to the trial, which can include geography [159, 155, 158, 160] and regulatory considerations [155, 159].

###### Trial outcomes.

The trial outcome is a binary label y∈{1,0}, where y=1 indicates the trial met their primary endpoints, while 0 means failing to meet with the primary endpoints.

The learning task is to learn a model $f_{\theta }$ for predicting the trial success probability $\overset{ˆ}{y}$, where $\overset{ˆ}{y}\in [0,\;1]$:

(41)
$$\overset{ˆ}{y}=f_{\theta }\left(p\in P,d\in D,t\in T,c\in C\right).$$

##### TOP Dataset

TOP [8] consists of 17,538 clinical trials with 13,880 small-molecule drugs and 5,335 diseases. Out of these trials, 9,999 (57.0%) succeeded (i.e., meeting primary endpoints), and 7,539 (43.0%) failed. For each clinical trial, we produce the following four data items: (1) drug molecule information, including Simplified Molecular Input Line Entry System (SMILES) strings and molecular graphs for the drug candidates used in the trials; (2) disease information including ICD-10 codes (disease code), disease description, and disease hierarchy in terms of CCS codes (https://www.hcup-us.ahrq.gov/toolssoftware/ccs10/ccs10.jsp); (3) trial eligibility criteria are in unstructured natural language and contain inclusion and exclusion criteria; and (4) trial outcome information includes a binary indicator of trial success (1) or failure (0), trial phase, start and end date, sponsor, and trial size (i.e., number of participants).

###### Dataset statistics.

Phase I: 2,402 trials / Phase II: 7,790 trials / Phase III: 5,741 trials

Dataset split.

Temporal Split as defined in [8] and Section A.3.5

References. [8]

Dataset license.

Non-Commercial Use

#### C.2.5 Structure-Based Drug Design Problem Formulation

Structure-based Drug Design aims to generate diverse, novel molecules with high binding affinity to protein pockets (3D structures) and desirable chemical properties. These properties are measured by oracle functions. A machine learning task first learns the molecular characteristics given specific protein pockets from a large set of protein-ligand pair data. Then, from the learned conditional distribution, we can sample novel candidates.

##### Target candidate set.

The target candidate set includes proteins, nucleic acids, or other biomolecules drugs can interact with, producing a therapeutic effect or causing a biological response. It is denoted by:

(42)
$$T=\left\{t_{1},\ldots ,t_{N_{t}}\right\},$$

where $t_{1},\ldots ,t_{N_{t}}$ are $N_{t}$ target candidates for the evaluated set of drugs. Information modeled for target candidates can include interaction, structural, and sequence information.

##### Ligand candidate set.

The ligand drug candidate set includes the drug molecules being tested for a particular therapeutic effect or biological response. It is denoted by:

(43)
$$L=\left\{l_{1},\ldots ,l_{N_{l}}\right\},$$

where $l_{1},\ldots ,l_{N_{l}}$ are the $N_{l}$ ligand/drug molecules being evaluated. Drug modeling can include molecular structure, often represented in formats such as SMILES (Simplified Molecular Input Line Entry System) or InChI (International Chemical Identifier) [163], physicochemical properties like hydrophobicity and molecular weight [105], and molecular descriptors and fingerprints [164].

##### Scoring function.

The scoring function, denoted by S, evaluates the binding affinity of ligand l∈L to protein target t∈T.

##### Drug-likeness function.

Function representing the drug-likeness of ligand l∈L, including properties like solubility, stability, and toxicity.

The generative learning task is to generate the ligand l∈L maximizing binding affinity, $S_{\theta }$, and drug-likeness, $f_{\theta }$. Given a loss function, Loss(S(t,l),f(l)), for t∈T and l∈L, the first step is to learn a model $M_{\theta }$ s.t.,

(44)
$$M_{\theta }={\mathrm{argmin}}_{\theta }\left[\mathrm{Loss}\left(S_{\theta }\left(t,l\right),f_{\theta }\left(l\right)\right)\right].$$

This is followed by the ligand optimization step, which optimizes the ligand for maximum binding affinity and drug-likeness given the trained model. A ligand optimization function, F, such as addition or multiplication, is used for the optimization:

(45)
$$l^{*}={\mathrm{argmax}}_{l\in L}\left[F\left(S_{\theta }\left(t\in T,\;l\right),{\;f}_{\theta }\left(l\right)\right)\right].$$

An example formulation would be as follows:

(46)
$$l^{*}={\mathrm{argmax}}_{l\in L}\left[S_{\theta }\left(t\in T,l\right)\times f_{\theta }\left(l\right)\right].$$

###### PDBBind Dataset

PDBBind is a comprehensive database extracted from PDB with experimentally measured binding affinity data for protein-ligand complexes. PDBBind does not allow the dataset to be re-distributed in any format. Thus, we could not host it on the TDC server. However, we provide an alternative route since significant processing is required to prepare the dataset ML. The user only needs to register at http://www.pdbbind.org.cn/, download the raw dataset, and then provide the local path. TDC will then automatically detect the path and transform it into an ML-ready format for the TDC data loader.

##### Dataset statistics.

19,445 protein-ligand pairs

Dataset split.

Random Split

References. [5]

###### Dataset license.

See note in the description on the TDC website

##### DUD-E Dataset

DUD-E provides a directory of valuable decoys for protein-ligand docking.

###### Dataset statistics.

22,886 active compounds and affinities against 102 targets. DUD-E does not support pocket extraction as protein and ligand are not aligned.

Dataset split.

Random Split

References. [10]

Dataset license.

Not specified

##### scPDB Dataset

scPDB is processed from PDB for structure-based drug design that identifies suitable binding sites for protein-ligand docking.

###### Dataset statistics.

16,034 protein-ligand pairs over 4,782 proteins and 6,326 ligands

Dataset split.

Random Split

References. [11]

Dataset license.

Not specified
