# Supplementary material for: Signals in the Cells: Multimodal and Contextualized Machine Learning Foundations for Therapeutics
Source: bioRxiv. 2024 Nov 12:2024.06.12.598655. Preprint. [Version 3] doi: 10.1101/2024.06.12.598655 (PMC11212894; doi:10.1101/2024.06.12.598655)
Supplement: 1 [file NIHPP2024.06.12.598655V3-supplement-1.pdf]

bioRxiv preprint doi: https://doi.org/10.1101/2024.06.12.598655; this version posted November 12, 2024. The copyright holder for this preprint (which was not certified by peer review) is the author/funder, who has granted bioRxiv a license to display the preprint in perpetuity. It is made available under aCC-BY 4.0 International license.

7 Supplementary

7.1 Datasets

The website `tdcommons.ai` contains all datasets discussed in this manuscript under their corresponding tasks. These are: TDC.scDTI (`https://tdcommons.ai/multi_pred_tasks/scdti/`), TDC.PerturbOutcome (`https://tdcommons.ai/multi_pred_tasks/counterfactual/`), TDC.TCREpitope (`https://tdcommons.ai/multi_pred_tasks/tcrepitope/`), TDC.TrialOutcome (`https://tdcommons.ai/multi_pred_tasks/trialoutcome/`), TDC.SBDD (`https://tdcommons.ai/generation_tasks/sbdd/`). In addition, all TDC datasets are made available via the harvard dataverse `https://dataverse.harvard.edu/dataset.xhtml?persistentId=doi:10.7910/DVN/21LKWG`. Here we include dataset curation details and code for accessing all datasets for the introduced tasks.

7.1.1 (Li, Michelle, et al.) Dataset

To curate target information for a therapeutic area, we examine the drugs indicated for the therapeutic area of interest and its descendants. The two therapeutic areas examined are rheumatoid arthritis (RA) and inflammatory bowel disease. For rheumatoid arthritis, we collected therapeutic data (i.e., targets of drugs indicated for the therapeutic area) from OpenTargets for rheumatoid arthritis (EFO 0000685), ankylosing spondylitis (EFO 0003898), and psoriatic arthritis (EFO 0003778). For inflammatory bowel disease, we collected therapeutic data for ulcerative colitis (EFO 0000729), collagenous colitis (EFO 1001293), colitis (EFO 0003872), proctitis (EFO 0005628), Crohn's colitis (EFO 0005622), lymphocytic colitis (EFO 1001294), Crohn's disease (EFO 0000384), microscopic colitis (EFO 1001295), inflammatory bowel disease (EFO 0003767), appendicitis (EFO 0007149), ulcerative proctosigmoiditis (EFO 1001223), and small bowel Crohn's disease (EFO 0005629).

bioRxiv preprint doi: https://doi.org/10.1101/2024.06.12.598655; this version posted November 12, 2024. The copyright holder for this preprint (which was not certified by peer review) is the author/funder, who has granted bioRxiv a license to display the preprint in perpetuity. It is made available under aCC-BY 4.0 International license.

We define positive examples (i.e., where the label y = 1) as proteins targeted by drugs that have at least completed phase 2 of clinical trials for treating a specific therapeutic area. As such, a protein is a promising candidate if a compound that targets the protein is safe for humans and effective for treating the disease. We retain positive training examples activated in at least one cell type-specific protein interaction network.

We define negative examples (i.e., where the label y = 0) as druggable proteins that do not have any known association with the therapeutic area of interest according to Open Targets. A protein is deemed druggable if targeted by at least one existing drug. We extract drugs and their nominal targets from Drugbank. We retain negative training examples activated in at least one cell type-specific protein interaction network.

Dataset statistics. The final number of positive (negative) samples for RA and IBD were 152 (1,465) and 114 (1,377), respectively. In [4], this dataset was augmented to include 156 cell types.

Dataset split. Cold Split: We split the dataset such that about 80% of the proteins are in the training set, about 10% of the proteins are in the validation set, and about 10% of the proteins are in the test set. The data splits are consistent for each cell type context to avoid data leakage.

References. [4]

Dataset license. CC BY 4.0

Code Sample

The dataset and splits are currently available on TDC Harvard Dataverse. In addition, you may obtain the protein splits used in [4] via the following code.

```
from tdc.resource.dataloader import DataLoader
data = DataLoader(name="opentargets_dti")
splits = data.get_split()
```

7.1.2 scPerturb Dataset

The scPerturb dataset is a comprehensive collection of single-cell perturbation data harmonized to facilitate the development and benchmarking of computational methods in systems biology. It includes various types of molecular readouts, such as transcriptomics, proteomics, and epigenomics. scPerturb is a harmonized dataset that compiles single-cell perturbation-response data. This dataset is designed to support the development and validation of computational tools by providing a consistent and comprehensive resource. The data includes responses to various genetic and chemical perturbations, crucial for understanding cellular mechanisms and developing therapeutic strategies. Data from different sources are uniformly pre-processed to ensure consistency. Rigorous quality control measures are applied to maintain high data quality. Features across different datasets are standardized for easy comparison and integration.

Dataset statistics. 44 publicly available single-cell perturbation-response datasets. Most datasets have, on average, approximately 3000 genes measured per cell. 100,000+ perturbations.

Dataset split. Cold Split and **Random Split** defined on cell lines and perturbation types.

References. [69]

Dataset license. CC BY 4.0

Code Sample

```
from tdc.multi_pred.perturboutcome import PerturbOutcome
from pandas import DataFrame
test_loader = PerturbOutcome(
    name="scperturb_drug_AissaBenevolenskaya2021")
testdf = test_loader.get_data()
```

bioRxiv preprint doi: https://doi.org/10.1101/2024.06.12.598655; this version posted November 12, 2024. The copyright holder for this preprint (which was not certified by peer review) is the author/funder, who has granted bioRxiv a license to display the preprint in perpetuity. It is made available under aCC-BY 4.0 International license.

7.1.3 TCHard Dataset

The TChard dataset is designed for TCR-peptide/-pMHC binding prediction. It includes over 500,000 samples from sources such as IEDB, VDJdb, McPAS-TCR, and the NetTCR-2.0 repository. The dataset is utilized to investigate how state-of-the-art deep learning models generalize to unseen peptides, ensuring that test samples include peptides not found in the training set. This approach highlights the challenges deep learning methods face in robustly predicting TCR recognition of peptides not previously encountered in training data.

Dataset statistics. 500,000 samples

Dataset split. Cold Split referred to as "Hard" split in [90].

References. [90]

Dataset license. Non-Commercial Use

Code Sample

```
from tdc.resource.dataloader import DataLoader
data = DataLoader(name="tchard")
self.split = data.get_split()
```

7.1.4 PanPep Dataset

PanPep is a framework constructed in three levels for predicting the peptide and TCR binding recognition. We have provided the trained meta learner and external memory, and users can choose different settings based on their data available scenarios: Few known TCRs for a peptide: few-shot setting; No known TCRs for a peptide: zero-shot setting; plenty of known TCRs for a peptide: majority setting. More information is available in the Github repo .

Dataset statistics. Data from multiple studies involving millions of TCR sequences.

Dataset split. Cold Split referred to as "Hard" split in [91].

References. [91]

Dataset license. GPL-3.0

Code Sample

```
from tdc.resource.dataloader import DataLoader
data = DataLoader(name="panpep")
self.split = data.get_split()
```

7.1.5 (Ye X et al) Dataset

Affinity selection-mass spectrometry data of discovered ligands against single biomolecular targets (MDM2, ACE2, 12ca5) from the Pentelute Lab of MIT This dataset contains affinity selection-mass spectrometry data of discovered ligands against single biomolecular targets. Several AS-MS-discovered ligands were taken forward for experimental validation to determine the binding affinity (KD) as measured by biolayer interferometry (BLI) to the listed target protein. If listed as a "putative binder," AS-MS alone was used to isolate the ligands to the target, with KD < 1 uM required and often observed in orthogonal assays, though there is some (< 50%) chance that the ligand is nonspecific. Most of the ligands are putative binders, with 4446 total provided. For those characterized by BLI (only 34 total), the average KD is 266 ± 44 nM; the median KD is 9.4 nM.

Dataset statistics. 34 positive ligands, 4446 putative binders, and three proteins

Dataset Split. Stratified Split and **N/A Split**: We provide stratified 10/90 split on train/test as well as "test set only" split.

References. [106, 15]

Dataset license. CC BY 4.0

Code Sample

bioRxiv preprint doi: https://doi.org/10.1101/2024.06.12.598655; this version posted November 12, 2024. The copyright holder for this preprint (which was not certified by peer review) is the author/funder, who has granted bioRxiv a license to display the preprint in perpetuity. It is made available under aCC-BY 4.0 International license.

```
from tdc.multi_pred import ProteinPeptide
data = ProteinPeptide(name="brown_mdm2_ace2_12ca5")
data.get_split()
```

7.1.6 TOP Dataset

TOP [14] consists of 17,538 clinical trials with 13,880 small-molecule drugs and 5,335 diseases. Out of these trials, 9,999 (57.0%) succeeded (i.e., meeting primary endpoints), and 7,539 (43.0%) failed. For each clinical trial, we produce the following four data items: (1) drug molecule information, including Simplified Molecular Input Line Entry System (SMILES) strings and molecular graphs for the drug candidates used in the trials; (2) disease information including ICD-10 codes (disease code), disease description, and disease hierarchy in terms of CCS codes (https://www.hcup-us.ahrq.gov/toolssoftware/ccs10/ccs10.jsp); (3) trial eligibility criteria are in unstructured natural language and contain inclusion and exclusion criteria; and (4) trial outcome information includes a binary indicator of trial success (1) or failure (0), trial phase, start and end date, sponsor, and trial size (i.e., number of participants).

Dataset statistics. Phase I: 2,402 trials / Phase II: 7,790 trials / Phase III: 5,741 trials.

Dataset split. Temporal Split as defined in [14] and Section 6.4.1.

References. [14]

Dataset license. Non-Commercial Use

Code Sample

```
from tdc.multi_pred import TrialOutcome
data = TrialOutcome(name = 'phase1') # 'phase2' / 'phase3'
split = data.get_split()
```

7.1.7 PDBBind Dataset

PDBBind is a comprehensive database extracted from PDB with experimentally measured binding affinity data for protein-ligand complexes. PDBBind does not allow the dataset to be re-distributed in any format. Thus, we could not host it on the TDC server. However, we provide an alternative route since significant processing is required to prepare the dataset ML. The user only needs to register at http://www.pdbbind.org.cn/, download the raw dataset, and then provide the local path. TDC will then automatically detect the path and transform it into an ML-ready format for the TDC data loader.

Dataset statistics. 19,445 protein-ligand pairs

Dataset split. Random Split

References. [107]

Dataset license. See note in the description on the TDC website.

Code Sample

```
from tdc.generation import SBDD
data = SBDD(name='PDBBind', path='./pdbbind')
split = data.get_split()
```

DUD-E Dataset

DUD-E provides a directory of valuable decoys for protein-ligand docking.

Dataset statistics. 22,886 active compounds and affinities against 102 targets. DUD-E does not support pocket extraction as protein and ligand are not aligned.

Dataset split. Random Split

References. [109]

Dataset license. Not specified

bioRxiv preprint doi: https://doi.org/10.1101/2024.06.12.598655; this version posted November 12, 2024. The copyright holder for this preprint (which was not certified by peer review) is the author/funder, who has granted bioRxiv a license to display the preprint in perpetuity. It is made available under aCC-BY 4.0 International license.

Code Sample

```
from tdc.generation import SBDD
data = SBDD(name='dude')
split = data.get_split()
```

7.1.8 scPDB Dataset

scPDB is processed from PDB for structure-based drug design that identifies suitable binding sites for protein-ligand docking.

Dataset statistics. 16,034 protein-ligand pairs over 4,782 proteins and 6,326 ligands

Dataset split. Random Split

References. [108]

Dataset license. Not specified

Code Sample

```
from tdc.generation import SBDD
data = SBDD(name='scPDB')
split = data.get_split()
```

7.2 Equations

7.2.1 `TDC.scDTI`: Contextualized Drug-Target Nomination (Identification)

TDC-2 introduces `TDC.scDTI` task. The predictive, non-generative task is formalized as learning an estimator for a disease-specific function f of a target protein and cell type outputting whether the candidate protein t is a therapeutic target in that cell type c:

$$y = f(t, c). \tag{1}$$

Target candidate set. The target candidate set includes proteins, nucleic acids, or other molecules drugs can interact with, producing a therapeutic effect or causing a biological response. The target candidate set is constrained to proteins relevant to the disease being treated. It is denoted by:

$$\mathbb{T} = \{t_1, \ldots, t_{N_t}\}, \tag{2}$$

where $t_1, \ldots, t_{N_t}$ are N_t target candidates for the drugs treating the disease. Information modeled for target candidates can include interaction, structural, and sequence information.

Biological context set. The biological context set includes the cell-type-specific contexts in which the target candidate set operates. This set is denoted as:

$$\mathbb{C} = \{c_1, \ldots, c_{N_c}\}, \tag{3}$$

where $c_1, \ldots, c_{N_c}$ are N_c biological contexts on which drug-target interactions are being evaluated. Information modeled for cell-type-specific biological contexts can include gene expression and tissue hierarchy. The set is constrained to disease-specific cell types and tissues.

Drug-target identification. Drug-Target Identification is a binary label $y \in \{1, 0\}$, where $y = 1$ indicates the protein is a candidate therapeutic target. At the same time, 0 means the protein is not such a target.

The goal is to train a model f_θ for predicting the probability $\hat{y} \in [0, 1]$ that a protein is a candidate therapeutic target in a specific cell type. The model learns an estimator for a disease-specific function of a protein target $t \in \mathbb{T}$ and a cell-type-specific biological context $c \in \mathbb{C}$ as input, and the model is tasked to predict:

$$\hat{y} = f_\theta(t \in \mathbb{T}, c \in \mathbb{C}). \tag{4}$$

bioRxiv preprint doi: https://doi.org/10.1101/2024.06.12.598655; this version posted November 12, 2024. The copyright holder for this preprint (which was not certified by peer review) is the author/funder, who has granted bioRxiv a license to display the preprint in perpetuity. It is made available under aCC-BY 4.0 International license.

7.2.2 `TDC.PerturbOutcome`: Perturbation-Response Problem Formulation

TDC-2 introduces Perturbation-Response prediction task. The predictive, non-generative task is formalized as learning an estimator for a function of the cell-type-specific gene expression response to a chemical or genetic perturbation, taking a perturbation $p \in \mathbb{P}$, a pre-perturbation gene expression profile from the control set $e_0 \in \mathbb{E}_{\not{p}}$, and the biological context $c \in \mathbb{C}$ under which the gene expression response to the perturbation is being measured:

$$y = f(p, e_0, c). \tag{5}$$

We center our definition on regression for the cell-type-specific gene expression vector in response to a chemical or genetic perturbation.

Perturbation set. The perturbation set includes genetic and chemical perturbations. It is denoted by:

$$\mathbb{P} = \{p_1, \ldots, p_{N_p}\}, \tag{6}$$

where $t_p, \ldots, p_{N_p}$ are N_p evaluated perturbations. Information modeled for genetic perturbations can include the type of perturbation (i.e., knockout, knockdown, overexpression) and target gene(s) of the perturbation. Information modeled for chemical perturbations can include chemical structure (i.e., SMILES, InChl) and concentration and duration of treatment.

Control set. The control set includes the unperturbed gene expression profiles. This set is denoted as:

$$\mathbb{E}_{\not{p}} = \{\vec{e}_{0_1}, \ldots, \vec{e}_{N_{e_0}}\}, \tag{7}$$

where $\vec{e}_{0_1}, \ldots, \vec{e}_{N_{e_0}}$ are N_{e_0} unperturbed gene expression profile vectors. Information models for gene expression profiles can include raw or normalized gene expression counts, transcriptomic profiles, and isoform-specific expression levels.

Biological context set. The biological context set includes the cell-type-specific contexts under which the perturbed gene expression profile is measured. It is denoted by:

$$\mathbb{C} = \{c_1, \ldots, c_{N_c}\}, \tag{8}$$

where $c_1, \ldots, c_{N_c}$ are the N_c biological contexts under which perturbations are being evaluated. Information modeled for biological contexts can include cell type or tissue type and experimental conditions [69] as well as epigenetic markers [121, 122].

Perturbation-response readouts. Perturbation-Response is a gene expression vector $\vec{e_1}$, where $\vec{e_1}_i$ denotes the expression of the i-th gene in the vector. It is the outcome of applying a perturbation, $p_i \in \mathbb{P}$, within a biological context, $c_j \in \mathbb{C}$, to a cell with a measured control gene expression vector, $e_{0_k} \in \mathbb{E}_{\not{p}}$.

The Perturbation-Response Prediction learning task is to learn a regression model f_θ estimating the perturbation-response gene expression vector $\hat{\vec{e_1}}$ for a perturbation applied in a cell-type-specific biological context to a control:

$$\hat{\vec{e_1}} = f_\theta(p \in \mathbb{P}, e_0 \in \mathbb{E}_{\not{p}}, c \in \mathbb{C}). \tag{9}$$

7.2.3 `TDC.ProteinPeptide`: Protein-Peptide Interaction Prediction Problem Formulation

TDC-2 introduces the Protein-Peptide Binding Affinity prediction task. The predictive, non-generative task is to learn a model estimating a function of a protein, peptide, antigen processing pathway, biological context, and interaction features. It outputs a binding affinity value (e.g., dissociation constant Kd, Gibbs free energy ΔG) or binary label indicating strong or weak binding. The binary label can also include additional biomarkers, such as allowing for a positive label if and only if the binding interaction is specific [15, 123, 124]. To account for additional biomarkers beyond binding affinity value, our task is specified with a binary label.

Protein set. The protein set includes target proteins. It is denoted by:

$$\mathbb{P} = \{p_1, \ldots, p_{N_p}\}, \tag{10}$$

where $p_1, \ldots, p_{N_p}$ are N_p target proteins. Information modeled for proteins can include sequence, structural, or post-translational modification data.

Peptide set. The control set includes the peptide candidates. This set is denoted as:

$$\mathbb{S} = \{s_1, \ldots, s_{N_s}\}, \tag{11}$$

bioRxiv preprint doi: https://doi.org/10.1101/2024.06.12.598655; this version posted November 12, 2024. The copyright holder for this preprint (which was not certified by peer review) is the author/funder, who has granted bioRxiv a license to display the preprint in perpetuity. It is made available under aCC-BY 4.0 International license.

where $s_1, \ldots, s_{N_s}$ are N_s candidate peptides. Information modeled for candidate peptides can include sequence, structural, and physicochemical data.

Antigen processing pathway set. The antigen processing pathway set includes antigen processing pathway profile information about prior steps in the biological antigen presentation pathway processes. It is denoted by:

$$\mathbb{A} = \{a_1, \ldots, a_{N_a}\}, \tag{12}$$

where $a_1, \ldots, a_{N_a}$ are the N_a antigen processing pathway profiles modeled. Information modeled in a profile can include proteasomal cleavage sites [125], classification into viral, bacterial, and self-protein sources and endogenous vs exogenous processing pathway [126, 127, 84, 128], and target/receptor-specific pathway attributes such as transporter associated with antigen processing (TAP) affinity [129], and endosomal/lysosomal processing efficiency [130].

Interaction set. It contains the interaction feature profiles. The set is denoted by:

$$\mathbb{I} = \{i_1, \ldots, i_{N_i}\}, \tag{13}$$

where $i_1, \ldots, i_{N_i}$ are the N_i interaction feature profiles. Information modeled in an interaction feature profile can include contact maps [131, 132, 133, 134], distance maps [132, 135], electrostatic interactions [131], and hydrogen bonds [131].

Cell-type-specific biological context set. It contains the interaction feature profiles. The set is denoted by:

$$\mathbb{C} = \{c_1, \ldots, c_{N_c}\}, \tag{14}$$

where $c_1, \ldots, c_{N_c}$ are the N_c cell-type-specific biological contexts under which the protein-peptide interaction is being evaluated. Information modeled in the cell-type-specific biological context can include transcriptomic and proteomic data. We note, however, that, to our knowledge, single-cell transcriptomic and proteomic data has yet to be used in protein-peptide binding affinity prediction, outlining a promising avenue of research in developing machine learning models for peptide-based therapeutics.

Protein-peptide interaction. It is a binary label, $y \in \{1, 0\}$, where $y = 1$ indicates a protein-peptide pair met the target biomarkers and $y = 0$ indicates the pair did not meet the target biomarkers.

The Protein-Peptide Interaction Prediction learning task is to learn a binary classification model f_θ estimating the probability, $\hat{y}$, of a protein-peptide interaction meeting specific biomarkers:

$$\hat{y} = f_\theta(p \in \mathbb{P}, s \in \mathbb{S}, a \in \mathbb{A}, i \in \mathbb{I}, c \in \mathbb{C}). \tag{15}$$

7.2.4 Clinical Trial Outcome Prediction Problem Formulation

The Clinical Trial Outcome Prediction task is formulated as a binary classification problem, where the machine learning model predicts whether a clinical trial will have a positive or negative outcome. It is a function that takes patient data, trial design, treatment characteristics, disease, and macro variables as inputs and outputs a trial outcome prediction, a binary indicator of trial success (1) or failure (0).

Patient set. The patient set includes one or multiple patient sub-populations, with the extreme case representing personalization. It is denoted as follows:

$$\mathbb{P} = \{p_1, \ldots, p_{N_p}\}, \tag{16}$$

where $p_1, \ldots, p_{N_p}$ are N_p patient sub-populations in this trial. The TOP benchmark [14] dataset represents patient data as part of the trial eligibility criteria. Patient data can include demographics [136, 137, 138, 139, 140], baseline health metrics [139, 140, 141], and medical history [136, 137, 138, 139, 140].

Trial design set. The trial design set includes this clinical trial's design profiles. It is denoted as:

$$\mathbb{D} = \{d_1, \ldots, d_{N_d}\}, \tag{17}$$

where $d_1, \ldots, d_{N_d}$ are N_d eligible trial design profiles for this clinical trial. Trial design profiles can model information including phase of the trial [14], number of participants, duration of the trial, trial eligibility criteria [14], and randomization and blinding methods [142, 143, 144].

Treatment set. The treatment set includes the candidate treatments for the trial. It is denoted as:

$$\mathbb{T} = \{t_1, \ldots, t_{N_t}\}, \tag{18}$$

bioRxiv preprint doi: https://doi.org/10.1101/2024.06.12.598655; this version posted November 12, 2024. The copyright holder for this preprint (which was not certified by peer review) is the author/funder, who has granted bioRxiv a license to display the preprint in perpetuity. It is made available under aCC-BY 4.0 International license.

where $t_1, \ldots, t_{N_t}$ are N_t candidate treatments for the clinical trial. The information modeled for treatments can include type of treatment (drug [14, 145], device [146, 147, 148], procedure [149, 150, 151, 152, 153]), dosage and administration route [142, 141, 154], mechanism of action [155, 156, 157], pre-clinical and early-phase trial results [156, 141, 158, 159].

Macro context set. The macro context set contains the configurations of macro variables relevant to the clinical trial. It is denoted as:

$$\mathbb{C} = \{c_1, \ldots, c_{N_c}\}, \tag{19}$$

where $c_1, \ldots, c_{N_c}$ are N_c configurations containing the values for macro variables relevant to the trial, which can include geography [160, 156, 159, 161] and regulatory considerations [156, 160].

Trial outcomes. The trial outcome is a binary label $y \in \{1, 0\}$, where $y = 1$ indicates the trial met their primary endpoints, while 0 means failing to meet with the primary endpoints.

The learning task is to learn a model f_θ for predicting the trial success probability $\hat{y}$, where $\hat{y} \in [0, 1]$:

$$\hat{y} = f_\theta(p \in \mathbb{P}, d \in \mathbb{D}, t \in \mathbb{T}, c \in \mathbb{C}). \tag{20}$$

7.2.5 Structure-Based Drug Design Problem Formulation

Structure-based Drug Design aims to generate diverse, novel molecules with high binding affinity to protein pockets (3D structures) and desirable chemical properties. These properties are measured by oracle functions. A machine learning task first learns the molecular characteristics given specific protein pockets from a large set of protein-ligand pair data. Then, from the learned conditional distribution, we can sample novel candidates.

Target candidate set. The target candidate set includes proteins, nucleic acids, or other biomolecules drugs can interact with, producing a therapeutic effect or causing a biological response. It is denoted by:

$$\mathbb{T} = \{t_1, \ldots, t_{N_t}\}, \tag{21}$$

where $t_1, \ldots, t_{N_t}$ are N_t target candidates for the evaluated set of drugs. Information modeled for target candidates can include interaction, structural, and sequence information.

Ligand candidate set. The ligand drug candidate set includes the drug molecules being tested for a particular therapeutic effect or biological response. It is denoted by:

$$\mathbb{L} = \{l_1, \ldots, l_{N_l}\}, \tag{22}$$

where $l_1, \ldots, l_{N_l}$ are the N_l ligand/drug molecules being evaluated. Drug modeling can include molecular structure, often represented in formats such as SMILES (Simplified Molecular Input Line Entry System) or InChI (International Chemical Identifier) [162], physicochemical properties like hydrophobicity and molecular weight [80], and molecular descriptors and fingerprints [163].

Scoring function. The scoring function, denoted by S, evaluates the binding affinity of ligand $l \in \mathbb{L}$ to protein target $t \in \mathbb{T}$.

Drug-likeness function. Function representing the drug-likeness of ligand $l \in \mathbb{L}$, including properties like solubility, stability, and toxicity.

The generative learning task is to generate the ligand $l \in \mathbb{L}$ maximizing binding affinity, S_θ, and drug-likeness, f_θ. Given a loss function, $\text{Loss}(S(t, l), f(l))$, for $t \in \mathbb{T}$ and $l \in \mathbb{L}$, the first step is to learn a model M_θ s.t.,

$$M_\theta = \text{argmin}_\theta[\text{Loss}(S_\theta(t, l), f_\theta(l))]. \tag{23}$$

This is followed by the ligand optimization step, which optimizes the ligand for maximum binding affinity and drug-likeness given the trained model. A ligand optimization function, F, such as addition or multiplication, is used for the optimization:

$$l^* = \text{argmax}_{l \in \mathbb{L}}[F(S_\theta(t \in \mathbb{T}, l), f_\theta(l))]. \tag{24}$$

An example formulation would be as follows:

$$l^* = \text{argmax}_{l \in \mathbb{L}}[S_\theta(t \in \mathbb{T}, l) \times f_\theta(l)]. \tag{25}$$

bioRxiv preprint doi: https://doi.org/10.1101/2024.06.12.598655; this version posted November 12, 2024. The copyright holder for this preprint (which was not certified by peer review) is the author/funder, who has granted bioRxiv a license to display the preprint in perpetuity. It is made available under aCC-BY 4.0 International license.

7.2.6 Context-Specific Metrics

Context-specific metrics are defined to measure model performance at critical biological slices, with our benchmarks focused on measuring cell-type-specific model performance. For single-cell drug-target nomination, we measure model performance at top-performing cell types. The metrics chosen were: APR@5 Top-20 CT - average precision and recall at $k = 5$ for the 20 best-performing cell types (CT); AUROC Top-1 CT - AUROC for top-performing cell type; AUROC Top-10 CT and AUROC Top-20 CT - weighted average AUROC for top-10 and top-20 performing cell types, respectively, each weighted by the number of samples in each cell type. Formally, we define context-specific APR@5 and AUROC below.

Context-specific AUROC

To calculate the **AUROC for the top K performing cell types**, we first need to determine which cell types achieve the highest AUROC scores. After selecting the top-performing cell types, we weigh each top-performing cell type's AUROC score by the number of samples in that cell type.

We denote:

$$\mathbb{D} = \{(x_i, y_i, c_i)\}, \quad \forall i \in \mathbb{S} \tag{26}$$

Here, $\mathbb{D}$ denotes the dataset where x_i denotes the feature vector, y_i is the true label, and c_i is the cell type for sample i from $\mathbb{S}$. We further denote C, the set of unique cell types. Then, the AUROC for a specific cell type, $AUROC_c$, is computed as:

$$AUROC_c = AUROC(D_c) \tag{27}$$

Here, $D_c = \{(x_i, y_i)|c_i = c\}$ is the subset of the dataset for cell type c and $AUROC(D_c)$ represents the AUROC score computed over this subset. Once these are computed, values can be sorted in descending order to select the top X cell type with highest AUROC value.

$$C_K = \{c_1, c_2, \ldots, c_K\} \quad s.t. \quad AUROC_{c_i} \geq AUROC_{c_j}, \forall i \leq K, j > K \tag{28}$$

The weighted AUROC for the top K cell types is given by weighting each cell type's AUROC by the proportion of its samples relative to the total samples in the top K cell types.

$$AUROC_{TopK} = \frac{\sum_{c \in C_K} AUROC_c \times |D_c|}{\sum_{c \in C_K} |D_c|} \tag{29}$$

This measure represents a balance between representation and performance of the cell types.

Context-specific Average Precision at rank R (AP@R)

In our study, we let $R = 5$ and compute **AP@5 for the top K performing cell types**. We denote dataset and samples as above.

$$\mathbb{D} = \{(x_i, y_i, c_i)\}, \quad \forall i \in \mathbb{S} \tag{30}$$

Here, $\mathbb{D}$ denotes the dataset where x_i denotes the feature vector, y_i is the true label, and c_i is the cell type for sample i from $\mathbb{S}$. We further denote C, the set of unique cell types. The samples of each cell type, $D_c = (x_i, y_i)|c_i = c$, can be sorted based on the score output by the model for said sample $f(x_i)$, with average precision at rank type computed accordingly.

$$D_c^5 = \{x_1, \ldots, x_5\} \quad s.t. \quad f(x_i) \geq f(x_j), \forall i \leq 5, j > 5, c_i = c, c_j = c \tag{31}$$

$$AP@5_c = AP(\{y_1, \ldots, y_5\}, \{f(x_1), \ldots, f(x_5)\}), \quad x_i \in D_c^5 \tag{32}$$

The average precision at rank k at Top X cell types can then be defined as:

$$C_K = \{c_1, c_2, \ldots, c_k\} \quad s.t. \quad AP@5_{c_i} \geq AP@5_{c_j}, \forall i \leq K, j > K \tag{33}$$

$$AP@5_{TopK} = mean(\{AP@5_{c_i}\}, \quad \forall c_i \in C_K) \tag{34}$$

AP summarizes a precision-recall curve as the weighted mean of precisions achieved at each threshold, with the increase in recall from the previous threshold used as the weight. Some key advantages of using AP@K include robustness to (1) varied numbers of protein targets activated across cell type-specific protein interaction networks and (2) varied sizes of cell type-specific protein interaction networks [4]. We compute AP using the scikit package as specified in `https://scikit-learn.org/1.5/modules/generated/sklearn.metrics.average_precision_score.html`.

bioRxiv preprint doi: https://doi.org/10.1101/2024.06.12.598655; this version posted November 12, 2024. The copyright holder for this preprint (which was not certified by peer review) is the author/funder, who has granted bioRxiv a license to display the preprint in perpetuity. It is made available under aCC-BY 4.0 International license.

7.3 Algorithms, Program codes and Listings

We provide code samples for the components described in sections 6.3.1 and 6.3.2.

Listing 2: The above configuration augments a protein-peptide dataset with an additional modality, amino acid sequence, and invokes numerous data processing functions tailored to the specific needs of the underlying dataset. Added information for this demonstration can be found at: https://colab.research.google.com/drive/13MYlg5tWpywWbKYsJQXafKAlVF2hz-sP?usp=sharing. There are more complex workflows implemented for current TDC dataviews and all such views leveraging the DSL can be found in the repo at https://github.com/mims-harvard/TDC/blob/main/tdc/dataset_configs/config_map.py

```
from .config import DatasetConfig
from ..feature_generators.protein_feature_generator import
    ↪ ProteinFeatureGenerator


class BrownProteinPeptideConfig(DatasetConfig):
    """Configuration for the brown-protein-peptide datasets"""

    def __init__(self):
        super(BrownProteinPeptideConfig, self).__init__(
            dataset_name="brown_mdm2_ace2_12ca5",
            data_processing_class=ProteinFeatureGenerator,
            functions_to_run=[
                "autofill_identifier", "create_range", "
                    ↪ insert_protein_sequence"
            ],
            args_for_functions=[{
                "autofill_column": "Name",
                "key_column": "Sequence",
            }, {
                "column": "KD␣(nM)",
                "keys": ["Putative␣binder"],
                "subs": [0]
            }, {
                "gene_column": "Protein␣Target"
            }],
            var_map={
                "X1": "Sequence",
                "X2": "protein_or_rna_sequence",
                "ID1": "Name",
                "ID2": "Protein␣Target",
            },
        )
```

7.3.1 TDC-2 Multimodal Single-Cell Retrieval API

We focus on the use case of an ML researcher who wishes to train a model on a large-scale single-cell atlas. In particular, researchers would be familiar with and have trained models on traditional single-cell datasets such as Tabula Sapiens [51]. Their interest is to scale a model by training it on a more extensive single-cell atlas based on this reference dataset. We build such an API. Specifically, given a reference dataset available in CellXGene Discover [2], we allow the user to perform a memory-efficient query using TileDB-SOMA to expand the reference dataset to include cell entries with non-zero readouts for any of the genes present in the reference dataset. This allows users to build large-scale single-cell atlases on familiar reference datasets. The example below illustrates how a user may construct a large-scale atlas with Tabula Sapiens as the reference dataset. Other use cases include augmenting datasets using knowledge graphs and cell-type-specific biomedical contexts. These capabilities are all powered by the Model-View-Controller framework (section 6.3.1).

bioRxiv preprint doi: https://doi.org/10.1101/2024.06.12.598655; this version posted November 12, 2024. The copyright holder for this preprint (which was not certified by peer review) is the author/funder, who has granted bioRxiv a license to display the preprint in perpetuity. It is made available under aCC-BY 4.0 International license.

Listing 3: The example below illustrates how a user may construct a large-scale atlas with Tabula Sapiens as the reference dataset using the TDC-2 CELLXGENE API.

```
from tdc.multi_pred.single_cell import CellXGene
dataloader = CellXGene(name="Tabula␣Sapiens␣-␣All␣Cells")
gen = dataloader.get_data(
    value_filter="tissue␣==␣'brain'␣and␣sex␣==␣'male'"
)
df = next(gen)
```

Listing 4: In addition to our TDC-2 DataLoader API implementation for the CellXGene RPC API, we provide a simplified wrapper over the CellXGene Census Discovery API, which allows users to perform remote procedure calls to fetch Cell Census data in more machine-learning-friendly formats like Pandas and Scipy. We also maintain support for the AnnData format. Users can query Cell Census counts as well as metadata using this API. The code sample below illustrates such usage.

```
from tdc.resource import cellxgene_census

# initialize Census Resource and query filters
resource = cellxgene_census.CensusResource()
cell_value_filter = "tissue␣==␣'brain'␣and␣sex␣==␣'male'"
cell_column_names = ["assay", "cell_type", "tissue"]

# Obtaining cell metadata from the cellxgene census in pandas format
obsdf = resource.get_cell_metadata(
    value_filter=cell_value_filter,
    column_names=cell_column_names,
    fmt="pandas")
```

7.3.2 PrimeKG Knowledge Graph

PrimeKG supports drug-disease prediction by including an abundance of 'indications,' 'contradictions', and 'off-label use' edges, which are usually missing in other knowledge graphs. We accompany PrimeKG's graph structure with text descriptions of clinical guidelines for drugs and diseases to enable multimodal analyses [22]. The code below depict example use cases of the TDC-2 PrimeKG API. Demonstrations are additionally available in `https://colab.research.google.com/drive/1kYH8nt3nW7tXYBPNcfYuDbWxGTqOEnWg?usp=sharing`.

Listing 5: We illustrate here example utilities for retrieving drug-target-disease associations using the TDC-2 PrimeKG API

```
from tdc.resource import PrimeKG

pkg = PrimeKG()
pkgdf = pkg.get_data()

def get_all_drug_evidence(disease):
    """given a disease, retrieve all drugs interacting with proteins
        ↪ relevant to disease"""
    prots = pkgdf[(pkgdf["relation"] == "disease_protein") & (pkgdf["x_name"
        ↪ ] == disease)]["y_name"].unique()
    drugs = pkgdf[(pkgdf["relation"] == "drug_protein") & (pkgdf["y_name"].
        ↪ isin(prots))]
    relations = drugs["display_relation"].unique()
    out = {}
    for rel in relations:
        out[rel] = drugs[drugs["display_relation"] == rel]["x_name"].unique
            ↪ ()
    return out
```

bioRxiv preprint doi: https://doi.org/10.1101/2024.06.12.598655; this version posted November 12, 2024. The copyright holder for this preprint (which was not certified by peer review) is the author/funder, who has granted bioRxiv a license to display the preprint in perpetuity. It is made available under aCC-BY 4.0 International license.

```
def get_all_associated_targets(disease):
    return pkgdf[(pkgdf["relation"] == "disease_protein") & (pkgdf["x_name"]
        ↪ == disease)][["y_name", "display_relation"]]

def get_disease_disease_associations(disease):
    return pkgdf[(pkgdf["relation"] == "disease_disease") & (pkgdf["x_name"]
        ↪ == disease)][["y_name", "display_relation"]]

def get_labels_from_evidence(disease):
    diseases = get_disease_disease_associations(disease)["y_name"]
    out = set()
    for d in diseases:
        targets = get_all_associated_targets(d)["y_name"].unique()
        out.update(targets)
    return list(out)

def all_diseases_by_keyword(kw):
    return pkgdf[(pkgdf["relation"] == "disease_protein") & (pkgdf["x_name"
        ↪ ].str.contains(kw, case=False, na=False))]["x_name"].unique()

if __name__ == "__main__":
    x = all_diseases_by_keyword("autism")
    [get_all_drug_evidence(d) for d in x]
    [get_all_associated_targets(d) for d in x]
    [get_disease_disease_associations(d) for d in x]
    print([get_labels_from_evidence(d) for d in x])
```

Listing 6: Here we illustrate combinig the TDC-2 PrimeKG API with the networkx module to retrieve drug repositioning opportunities.

```
import networkx as nx
from tdc.resource import PrimeKG

# Load the PrimeKG data
kg = PrimeKG()
data = kg.get_data()
data = data[data["relation"].str.contains("drug")]

# Create a graph from the knowledge graph data
G = nx.from_pandas_edgelist(data, 'x_id', 'y_name', edge_attr='relation')

# Example function to find repositioning opportunities for a given drug
def find_repositioning_opportunities(drug):
    neighbors = list(G.neighbors(drug))
    diseases = [node for node in neighbors if G[drug][node]['relation'] == '
        ↪ drug_protein']
    return diseases

# Find repositioning opportunities for a specific drug
drug_name = 'DB00945'
repositioning_opportunities = find_repositioning_opportunities(drug_name)
```

7.3.3 TDC-2 Model Server

The introduced model server is composed of the TDC-2 Model Hub and a set of utilities and endpoints for facilitating model inference and fine-tuning. TDC-2 introduces The Commons' HuggingFace Model Hub. It is a resource with pre-trained models, including geometric deep learning models, large language models, and other contextualized multimodal models for therapeutic tasks. The models can be fine-tuned using datasets in TDC-2 and be used for downstream tasks such as

bioRxiv preprint doi: https://doi.org/10.1101/2024.06.12.598655; this version posted November 12, 2024. The copyright holder for this preprint (which was not certified by peer review) is the author/funder, who has granted bioRxiv a license to display the preprint in perpetuity. It is made available under aCC-BY 4.0 International license.

implementations of multi-agent collaborative schemes [164] (i.e., expert consultants) our predictive therapeutic tasks [3, 19]. The model hub details and available models can be found at `https://huggingface.co/tdc`.

Listing 7: The below illustrates the basic functionality of the model hub to download a model and perform inference on a precdictive task as well as fine-tune the model

```
from tdc import tdc_hf_interface
tdc_hf = tdc_hf_interface("BBB_Martins-AttentiveFP")
# load deeppurpose model from this repo
dp_model = tdc_hf.load_deeppurpose('./data')
tdc_hf.predict_deeppurpose(dp_model, ['YOUR␣SMILES␣STRING'])
# fine-tune
dp_model.train(train, val, test) # for some defined splits
```

Listing 8: The below illustrates using the tdc model hub to download a foundation model [3]

```
from tdc import tdc_hf_interface
from transformers import BertModel
geneformer = tdc_hf_interface("Geneformer")
model = geneformer.load()
assert isinstance(model, BertModel), type(model)
```

Listing 9: Beyond downloading a foundation model [3], the model server facilitates model inference across a range of datasets. Below an example integrating the TDC-2 CellXGene API with the model server.

```
from tdc.resource import cellxgene_census
from tdc.model_server.tokenizers.geneformer import GeneformerTokenizer
from tdc import tdc_hf_interface
import torch

# query the CELLXGENE census
adata = self.resource.get_anndata(
var_value_filter=
"feature_id␣in␣['ENSG00000161798',␣'ENSG00000188229']",
obs_value_filter=
"sex␣==␣'female'␣and␣cell_type␣in␣['microglial␣cell',␣'neuron']",
column_names={
    "obs": [
        "assay", "cell_type", "tissue", "tissue_general",
        "suspension_type", "disease"
    ]
},
)

# tokenize gene expression vectors
tokenizer = GeneformerTokenizer()
x = tokenizer.tokenize_cell_vectors(adata,
                                    ensembl_id="feature_id",
                                    ncounts="n_measured_vars")

cells, _ = x

# load the model
geneformer = tdc_hf_interface("Geneformer")
model = geneformer.load()

"""
Custom pre-processing code can include padding and attention mask
    ↪ definitions.
```

bioRxiv preprint doi: https://doi.org/10.1101/2024.06.12.598655; this version posted November 12, 2024. The copyright holder for this preprint (which was not certified by peer review) is the author/funder, who has granted bioRxiv a license to display the preprint in perpetuity. It is made available under aCC-BY 4.0 International license.

```
"""

input_tensor = torch.tensor(cells)
out = []
for batch in input_tensor:
    # build an attention mask
    attention_mask = torch.tensor(
        [[x[0] != 0, x[1] != 0] for x in batch])
    # run batched inference
    out.append(model(batch, attention_mask=attention_mask))
```

7.3.4 Running TDC-2 Benchmarks

We provide code for replicating all introduced benchmarks and testing other model performance on all TDC-2 tasks. We include here snippets for all introduced benchmarks.

Listing 10: The below code illustrates how to retrieve the train, test, and val splits used for the TDC.scDTI benchmark

```
from tdc.benchmark_group import scdti_group
group = scdti_group.SCDTIGroup()
train_val = group.get_train_valid_split()
tst = group.get_test()["test"]
# train your model and test on the test set
group.evaluate(preds)
```

Listing 11: The below code illustrates how to retrieve the train, test, and val splits used for the TDC.PerturbOutcome chemical perturbation benchmark

```
from tdc.benchmark_group import counterfactual_group
group = counterfactual_group.CounterfactualGroup()
train, val = group.get_train_valid_split(remove_unseen=False)
test = group.get_test()
# train your model and test on the test set
group.evaluate(preds)
```

Listing 12: The below code illustrates how to retrieve the train, test, and val splits used for the TDC.PerturbOutcome genetic perturbation benchmark

```
from tdc.benchmark_group import geneperturb_group
group = geneperturb_group.GenePerturbGroup()
train_val = group.get_train_valid_split()
test = group.get_test()
# train your model and test on the test set
group.evaluate(preds)
```

Listing 13: The below code illustrates how to retrieve the train, test, and val splits used for the TDC.TCREpitope benchmark

```
from tdc.benchmark_group.tcrepitope_group import TCREpitopeGroup
group = TCREpitopeGroup()
train_val = group.get_train_valid_split()
test = group.get_test()
# train your model and test on the test set
group.evaluate(preds)
```

7.3.5 External Links - Reproducibility

Here, we include pointers to external resources to reproduce the results reported in this manuscript.

bioRxiv preprint doi: https://doi.org/10.1101/2024.06.12.598655; this version posted November 12, 2024. The copyright holder for this preprint (which was not certified by peer review) is the author/funder, who has granted bioRxiv a license to display the preprint in perpetuity. It is made available under aCC-BY 4.0 International license.

Model Benchmarking

TDC-2 Benchmarking Tooling Code for Chemical Perturbations, https://github.com/mims-harvard/TDC/blob/main/tdc/benchmark_group/counterfactual_group.py

TDC-2 Benchmarking Tooling Code for CRISPR-based Perturbations, https://github.com/mims-harvard/TDC/blob/main/tdc/benchmark_group/geneperturb_group.py

Reproducing Benchmark Results for Clinical Trial Outcome Prediction, https://github.com/futianfan/clinical-trial-outcome-prediction

Evaluating Cell-Type-Specific Context Metrics for PINNACLE Across 10 Seeds, https://colab.research.google.com/drive/1gjZIfmF2Gmz3Nqm1uGP79l0AmsPAvj_5?usp=sharing

Evaluating Cell-Type-Specific Context Metrics for PINNACLE Across 10 Seeds. Outputs Referenced in PINNACLE [4] and its reproducibility documentation, https://drive.google.com/drive/folders/1QX05afMekucbtjl_07ZxZhgnKVH3OXMk?usp=sharing

Code for reproducing PINNACLE results [4], https://github.com/mims-harvard/PINNACLE/tree/main/evaluate

Reproducing TCR-Epitope results. Code for Benchmarking models in Section 3.3.1. A bash script for each negative sampling method is included for each TCR-Epitope model, https://drive.google.com/drive/folders/1O7G_h_06VDABM6U_Xt7otXPazKOXTAG9?usp=sharing

Reproducing Chemical Perturbation results. Code for Benchmarking models in Section 3.2.2 chemical perturbation section. A run_chemical_sc.py Python script is included for each model. Default settings were used from each model's GitHub repository, https://drive.google.com/drive/folders/1RlBnRPmWFRQ6M_1EQ_FMwFb1Y8IjXoyC?usp=sharing

Leaderboards

TDC.PerturbOutcome Leaderboard, https://tdcommons.ai/benchmark/counterfactual_group/overview/

TDC.ProteinPeptide Leaderboard, https://tdcommons.ai/benchmark/proteinpeptide_group/overview/

TDC.scDTI Leaderboard, https://tdcommons.ai/benchmark/scdti_group/overview/
